# Short‐term gradient imperfections in high‐resolution EPI lead to Fuzzy Ripple artifacts

**DOI:** 10.1002/mrm.30489

**Published:** 2025-04-02

**Authors:** Laurentius (Renzo) Huber, Rüdiger Stirnberg, A. Tyler Morgan, David A. Feinberg, Philipp Ehses, Lasse Knudsen, Omer Faruk Gulban, Kenshu Koiso, Isabel Gephart, Stephanie Swegle, Susan G. Wardle, Andrew S. Persichetti, Alexander J. S. Beckett, Tony Stöcker, Nicolas Boulant, Benedikt A. Poser, Peter A Bandettini

**Affiliations:** ^1^ NIMH, NIH Bethesda Maryland USA; ^2^ German Center for Neurodegenerative Diseases (DZNE) Bonn Germany; ^3^ Helen Wills Neuroscience Institute, University of California, Berkeley Berkeley California USA; ^4^ Advanced MRI Technologies Sebastopol California USA; ^5^ Department of Cognitive Neuroscience, Faculty of Psychology and Neuroscience Maastricht University Maastricht The Netherlands; ^6^ Center of Functionally Integrative Neuroscience (CFIN), Department of Clinical Medicine Aarhus University Aarhus Denmark; ^7^ Sino‐Danish Center for Education and Research (SDC) University of Chinese Academy of Sciences Beijing China; ^8^ Brain Innovation Maastricht The Netherlands; ^9^ University Paris Saclay CEA, CNRS, NeuroSpin, BAOBAB Gif‐sur‐Yvette France

**Keywords:** 7 T acquisition, Fuzzy Ripples, layer‐fMRI, ventral brain

## Abstract

**Purpose:**

High‐resolution fMRI is a rapidly growing research field focused on capturing functional signal changes across cortical layers. However, the data acquisition is limited by low spatial frequency EPI artifacts; termed here as Fuzzy Ripples. These artifacts limit the practical applicability of acquisition protocols with higher spatial resolution, faster acquisition speed, and they challenge imaging in inferior regions of the brain.

**Methods:**

We characterize Fuzzy Ripple artifacts across commonly used sequences and distinguish them from conventional EPI Nyquist ghosts and off‐resonance effects. To investigate their origin, we employ dual‐polarity readouts.

**Results:**

Our findings indicate that Fuzzy Ripples are primarily caused by readout‐specific imperfections in k‐space trajectories, which can be exacerbated by short‐term eddy current, and by inductive coupling between third‐order shims and readout gradients. We also find that these artifacts can be mitigated through complex‐valued averaging of dual‐polarity EPI or by disconnecting the third‐order shim coils.

**Conclusion:**

The proposed mitigation strategies allow overcoming current limitations in layer‐fMRI protocols: Achieving resolutions beyond 0.8 mm is feasible, and even at 3T, we achieved 0.53 mm voxel functional connectivity mapping. Sub‐millimeter sampling acceleration can be increased to allow sub‐second TRs and laminar whole brain protocols with up to GRAPPA 8. Sub‐millimeter fMRI is achievable in lower brain areas, including the cerebellum.

## 1 INTRODUCTION

### 1.1 Layer‐fMRI and its acquisition challenges

Layer‐fMRI has significant potential for investigating neural information flow within and across brain systems. Knowing at which cortical layer neural activity occurs allows neuroscientists to determine whether activation modulations are driven by feed‐forward or feedback input, and whether neural circuits are involved in output versus input processes.

However, traditional layer‐fMRI data collection at submillimeter resolutions faces limitations due to EPI artifacts. Specifically, in high‐resolution protocols with low bandwidths, EPI ghosts arise from inconsistencies between odd and even echoes. While conventional Nyquist ghosting is addressed through a common two‐parameter phase correction method (only including 0th and first‐order corrections^1^, ^2^, ^3^) layer‐fMRI is also affected by additional higher‐order phase inconsistencies:

- Imperfections in gradient waveforms increase with higher gradient amplitude and slew rates. This is particularly challenging for EPI image quality when large ramp sampling factors and low bandwidths are used in layer‐fMRI protocols.
- The long echo train length in high‐resolution protocols allows phase inconsistencies to accumulate during the acquisition of large imaging matrices.
- Parallel imaging, which is crucial for efficient layer‐fMRI, is very sensitive to odd‐even artifacts and can amplify small residual EPI ghosting and phase errors within the GRAPPA/SENSE reconstruction.^4^

Due to these challenges, conventional layer‐fMRI acquisition protocols are often conservatively designed, with limited resolutions of approximately 0.8 mm and TR values around 3 s. This spatial resolution is barely sufficient to resolve activity across layer groups 300–1200 μm apart with Nyquist sampling.^5^ Additionally, FOV prescriptions are typically restricted to cover only the outer brain areas of the upper cortex. This is because:

- Many lower brain areas are located far from the receive RF‐coil elements, leading to high g‐factors and low SNR.
- Lower brain areas experience stronger B_0_ inhomogeneities, resulting in more severe EPI artifacts.
- Lower brain areas require large matrix sizes for acquisition, but shorter T_2_* values lead to increased signal decay and reduced SNR.

Among the 275 human layer‐fMRI papers published between 1997 and 2024, fewer than five focus on low ventral brain structures (source https://layerfmri.com/papers).

The goal of this study is to:

1. Characterize a significant limitation of high‐resolution fMRI, referred to as “Fuzzy Ripple artifacts”, that prevent researchers from advancing to use Cartesian EPI protocols with smaller voxels, shorter TRs, and coverage of lower brain areas. The word “Fuzzy” refers to the unique feature of the artifact. Namely that it manifests as a local ghost with a low spatial frequency envelope of phase interference patterns. The image errors are blurry.
2. Identify the primary cause of this artifact: k_x_‐specifc EPI trajectory imperfections. And then characterizing the exacerbation of Fuzzy Ripples with respect to a number of system imperfections: Including (i) gradient imperfections caused by inductive coupling between third‐order shim coils and readout gradients, (ii) interactions of relatively high GRAPPA accelerations while having trajectory imperfections.
3. Implement and test a potential mitigation strategy: complex‐valued averaging of dual‐polarity EPI readouts, and unplugging the third‐order shims.
4. Empirically evaluate whether this mitigation strategy enables neuroimagers to surpass the current resolution limits of conventional layer‐fMRI protocols, with respect to TR, voxel size, and imaging of lower brain areas. Our focus will be on practical aspects in the most commonly used sub‐millimeter fMRI sequences, specifically the 3D‐EPI from DZNE^6^ with and without VASO^7^ and, to some extent, the Multi‐Band C^2^P from CMRR^8^ on SIEMENS 7T scanners equipped with the vendor provided SC72 gradient coil (Siemens Healthineers, Forchheim, Germany) which incorporates third‐order shimming capabilities.

### 1.2 Fuzzy Ripple artifacts are relevant for the field of layer‐fMRI

**FIGURE 1:**
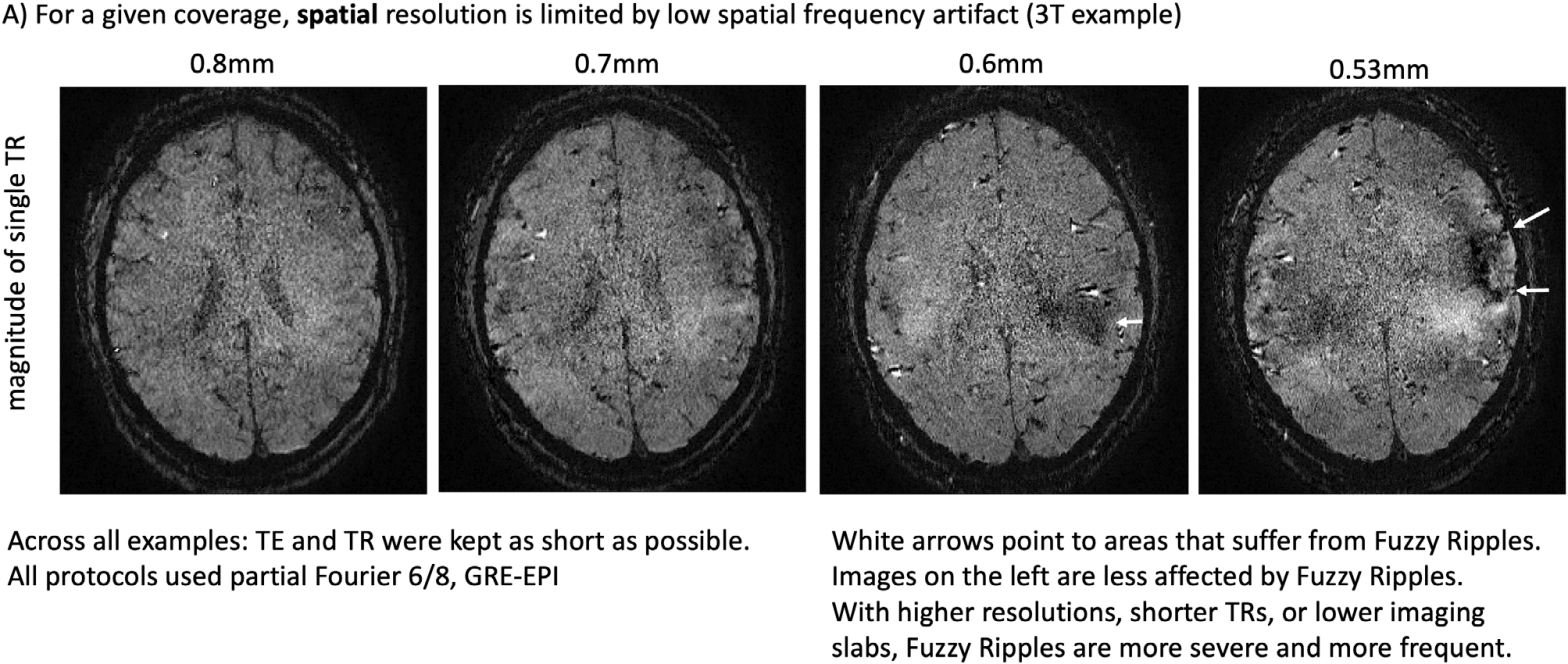
Fuzzy Ripples are the reason why layer‐fMRI is confined to conventional protocols. Fuzzy Ripples are the primary reason why layer‐fMRI is restricted to conventional protocols. Standard layer‐fMRI protocols are generally limited to 0.8 mm resolution with TRs of several seconds, focusing on upper cortical brain areas. These limitations cannot be surpassed because, with more ambitious acquisition protocols. Fuzzy Ripple artifacts become too strong and too frequent. The example shown here exemplifies issues of pushing resolutions beyond 0.8 mm. They refer to 3D‐EPI readouts with planar EPI trajectories. See Figure S1 for other sampling schemes of high‐resolution fMRI that amplify Fuzzy Ripple artifacts.

**FIGURE 2:**
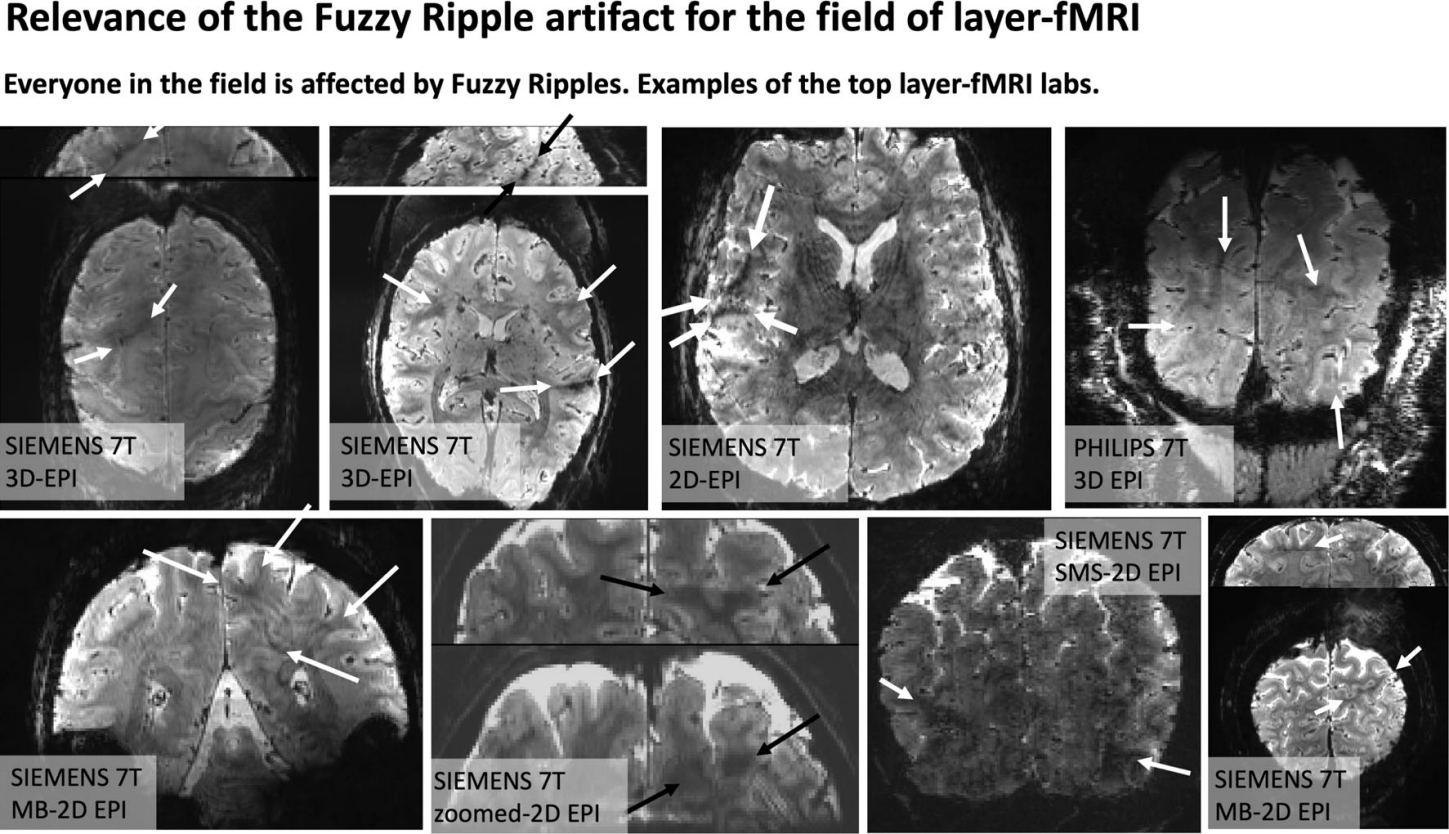
Fuzzy Ripples and their impact on layer‐fMRI research. The widespread effect of Fuzzy Ripples: Representative EPI images from the top 10 layer‐fMRI laboratories (based on number of publications on www.layerfmri.com/papers): Maastricht, Nijmegen/Essen, CMRR, NIH, MGH, Amsterdam, Leipzig, Cambridge. Note Utrecht/Tübingen are excluded, as despite being among the top 10 layer‐fMRI laboratories, none of their papers include publicly available layer‐fMRI EPI data.

Previously acquired EPI brain images, shown in Figures 1 and 2, highlight the types of imaging artifacts that pose limitations for high‐resolution fMRI. We refer to these artifacts as “Fuzzy Ripples”.

Figure 1 illustrates how the severity of these artifacts increases when conventional layer‐fMRI protocols —featuring 0.8 mm resolution— are pushed to smaller voxels (see Figure S1 for corresponding protocols pushing to shorter TRs and lower brain areas). While the conventional protocols (left column) may already show faint artifacts, the right columns demonstrate how these artifacts can become so severe that the data are rendered unusable. In the right column, it can be seen that these artifacts are the primary source of noise, more so than the thermal noise, which appears as a salt‐and‐pepper graininess in the images.

Although these artifacts are most pronounced in more aggressive protocols, they are also present in conventional protocols (0.8 mm, upper brain, few seconds TR), although usually at a lower magnitude that doesn't necessarily render the datasets unusable. Instead, they limit scanner operators' ability to achieve higher spatiotemporal resolutions across different brain areas in the first place. Figure 2 shows representative high‐resolution EPI data from the top 10 laboratories with the most published human layer‐fMRI papers. Without exception, Fuzzy Ripples are visible in all of them, underscoring the significance of this artifact for the entire research field.

Across all the EPI brain images shown in panels of Figures 1 and 2, artifacts share common characteristics. Specifically, they exhibit local signal intensity deviations at low spatial frequencies, which can be described as fuzzy clouds of brighter or darker signals. The darker Fuzzy Ripples are generally more noticeable to the naked eye. While these artifacts may contain wave‐like warble patterns at higher spatial frequencies, their overall appearance is usually blurry. The size of the artifacts typically ranges from 5% to 15% of the FOV. In this study, our goal is to identify the origins of Fuzzy Ripples and explore strategies for mitigating them.

## 2 THEORY

### 2.1 Readout direction specific eddy currents in ramp‐sampled EPI can result in odd‐even ghosts at low spatial frequencies

In conventional high‐resolution Cartesian EPI, as used in common 2D/Multiband‐EPI and 3D‐EPI, readout gradients are driven at their maximum allowed amplitude and slew rate. This operation pushes the gradients beyond the regime of vendor‐provided optimal eddy current compensation calibration.^9^

Furthermore, the design of conventional body gradients is not optimized for high‐resolution, encoding‐limited head EPI. As a result, the trapezoidal gradient pulse shapes heavily depend on data sampling along the slopes, with ramp sampling fractions reaching up to 78% of the entire duration of the read pulses. This is outside the range of conventional vendor‐provided EPI ghost correction methods, which typically assume that gradient delays can be corrected with line‐wise two‐parameter phase correction.^1^, ^2^, ^3^ With such high ramp sampling ratios, gradient delays introduce readout direction‐dependent phase offsets that vary significantly throughout the gradient pulse evolution.

**FIGURE 3:**
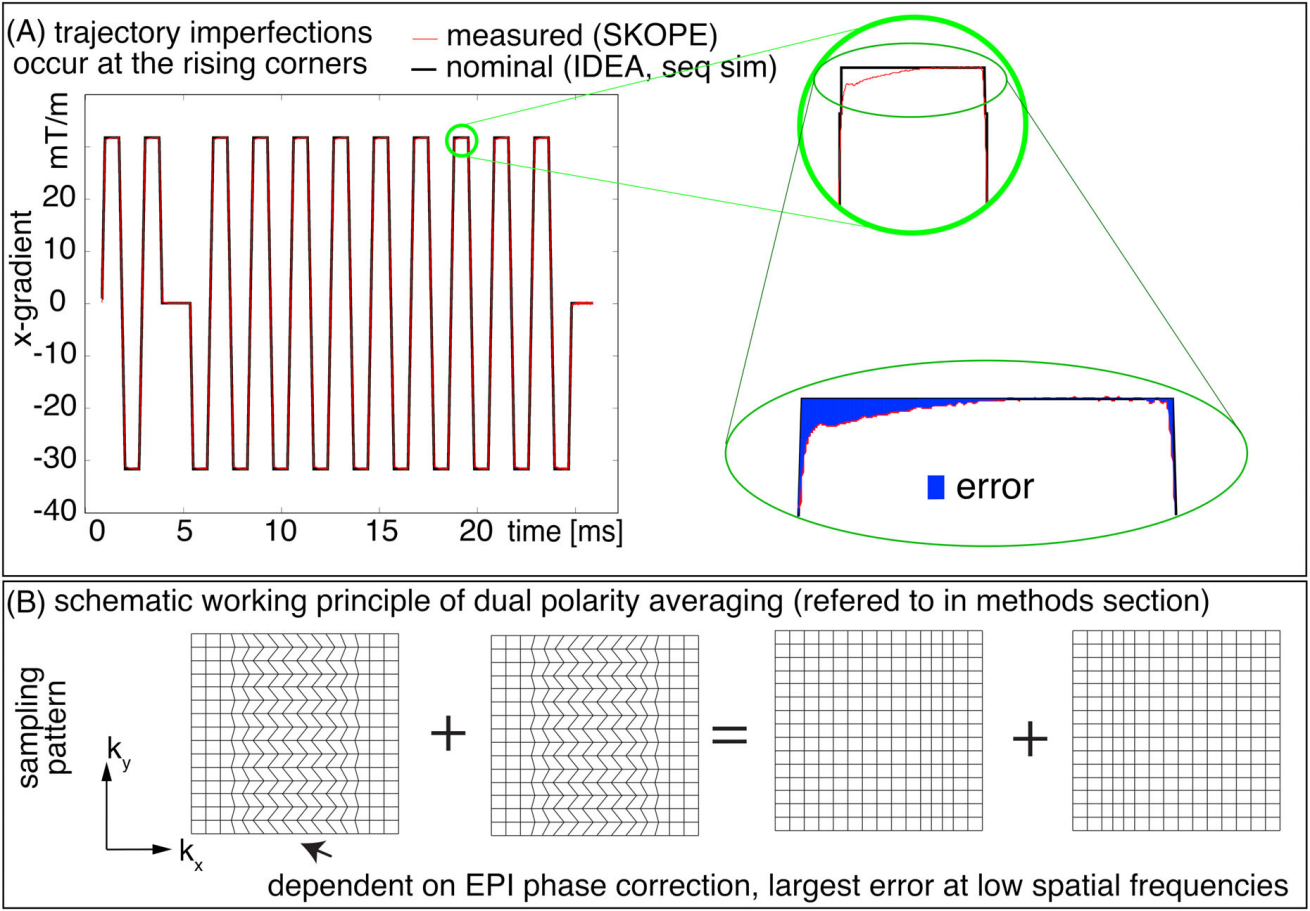
Concept of Fuzzy Ripples as EPI odd‐even delays with ramp sampling with readout (here k_x_)‐specific phase errors. (A) Gradient imperfections in high‐resolution EPI are most prominent at the corners of trapezoids, including both the rising and falling edges. The nominal EPI trajectory is derived from SIEMENS IDEA simulations, while the measured trajectory is obtained using SKOPE on a standard SIEMENS 7T MAGNETOM with third‐order shimming, at 0.8 mm resolution. (The full protocol parameters: https://github.com/layerfMRI/Sequence_Github/blob/master/Whole_brain_layers/20211012_KE). These data were acquired with a ramp‐sampling ratio of 72%. This means that the largest trajectory error of the trapezoid corners is 28% away from the center of k‐space. This section of the EPI sampling represents relatively low spatial frequencies of the image. First‐order SKOPE coefficients were used to generate trajectories of linear gradients. (B) Despite phase correction in the image reconstruction process that are designed for line‐by‐line delays of conventionally discussed odd‐even artifacts, residual imperfections persist in the part of k‐space that encodes lower spatial frequencies. These residual errors, shown as deviations in k_x_, manifest as irregular k‐space grids for odd and even lines. Such odd‐even errors are expected to produce EPI ghosting artifacts in low spatial frequencies. This study introduces a strategy to address Fuzzy Ripple artifacts by employing a dual‐polarity EPI approach that alternates the read direction every other TR (see Section 3). EPI images with opposite read directions are anticipated to produce ghosts with opposite phases.

Figure 3A illustrates the gradient shape and trajectory imperfections for a typical high‐resolution EPI protocol.^10^ It can be seen that the strongest gradient imperfections occur at the corners of the trapezoidal pulse shapes, which cannot be adequately corrected for by conventional vendor‐provided Nyquist ghost correction strategies. Instead, a global phase correction scheme may lead to residual phase errors that are distributed differently across representations of low and high spatial frequencies (Figure 3B). In this study, we hypothesized that these mechanisms may partially contribute to the Fuzzy Ripple artifacts.

The gradient trajectory imperfections visible in Figure 3 can be described as “short‐term eddy currents” in the range of 50–500 μs. While the vendor's tuneup procedure commonly quantifies five time constants of eddy currents in the typical range of 0.7 ms–5 s, short‐term eddy currents as visible in Figure 3 remain uncorrected in standard procedures.

### 2.2 Potential mitigation strategies of Fuzzy Ripples

#### 2.2.1 Dual‐polarity averaging

Combining dual‐polarity readouts with complex‐valued averaging on their respective images has been proposed as a method to reduce odd‐even artifacts of two forms: The odd‐even artifacts that arise from gradient delays and the odd‐even artifacts that arise from B_0_‐related off‐resonance effects.^11^, ^12^, ^13^, ^14^, ^15^, ^16^ These two forms of odd‐even artifacts are manifested as “edge‐ghosts” (high spatial frequency ghosts) in EPI‐based fMRI acquisitions. Different from those forms of odd‐even artifacts, the Fuzzy Ripple artifact is a higher‐order odd‐even artifact that arises from k_x_‐specific (non‐global) trajectory imperfections. Empirically, this higher‐order odd‐even artifact seems to be a dominant issue at higher resolutions and larger GRAPPA factors (Figures 1 and S1). However, also this higher‐order artifact can be addressed with the mitigation strategies that have been proposed for other odd‐even ghosts.

The principle of this approach is illustrated in Figure 3B. Reversed readout polarities are expected to completely invert the k‐space shift pattern, meaning that the resulting EPI ghosts should have opposite phases for each readout polarity and can be effectively canceled through complex‐valued averaging. While dual‐polarity averaging has previously been shown to mitigate off‐resonance induced artifacts in interleaved multi‐shot EPI,^15^ to some extent, it should be suited to mitigate further artifacts related to signal errors that switch signs or are opposedly shifted in k‐space with inverted readouts. In this study, we explored whether this strategy can also address low‐frequency ripple artifacts.

#### 2.2.2 Mitigation of eddy currents by minimizing inductive coupling between gradient and third‐order shim coils

Recent observations by Boulant et al.^17^ indicate that third‐order shim coils influence gradient‐magnet interactions. Due to the shared geometric symmetry of the third‐order shim coils and the gradients, inductive coupling can occur, leading to compromised gradient impulse response functions at certain frequencies. We investigated the impact of third‐order shims on Fuzzy Ripples and assessed whether disconnecting the third‐order shims could serve as a mitigation strategy.

## 3 METHODS

We scanned 38 participants as part of this study, all of whom provided informed consent. A total of 31 participants were scanned at the National Institutes of Health (NIH), while the remaining participants were scanned during the pilot phases at the University of Maastricht and the University of California, Berkeley, with each institution's local Institutional Review Board (IRB) approval, in accordance with the Declaration of Helsinki. Complete scanning protocols and sequence parameters are available here: https://github.com/layerfMRI/Sequence_Github/. Table 1 summarizes the key protocol parameters of the experiments described below.

**TABLE 1:** Overview of the key parameters in the experiments shown here.

Purpose	Figure	Echo spacing	Seq	T	Variable of interest	Other key parameter
Showing that Fuzzy Ripples are a substantial constraint for pushing to higher and higher resolutions.	1	0.5 mm–1.47 ms 0.6 mm–1.32 ms 0.7 mm–1.38 ms 0.8 mm–1.06 ms	3D‐EPI^6^	3	Resolution	GRAPPA 3, axial slab, TE = 34 ms, FOV = 169 mm
Showing that in high‐resolution protocols, there can be short‐term eddy currents at rising and falling corners of ramp sampling EPI.	3	0.98 ms	3D‐EPI^18^	7	Trajectory imperfections	GRAPPA 3, axial whole brain, TE = 24 ms, resolution = 0.8 mm, FOV = 130 mm
Showing how Fuzzy Ripples are different from other sources of odd‐even ghosts.	4	1.02 ms	3D‐EPI^6^	7	GRAPPA, Shim quality, Ramp sampling	GRAPPA 6 and 1, axial slab, TE = 26 ms, Ramp sampling on/off resolution = 1 mm FOV = 400 mm
Showing the effect of Fuzzy Ripples in activation maps.	5	1.0 ms	2D‐EPI^8^	7	Third‐order shim	GRAPPA 3, axial slab, TE = 26 ms, resolution = 0.8 mm FOV = 175 mm
Showing that Fuzzy Ripples can be mitigated with multiple independent approaches.	6	1.26 ms (unless otherwise stated)	3D‐EPI^6^	7	Echo spacing, third‐order shim, dual‐polarity averaging	GRAPPA 3, axial slab, TE = 25 ms, resolution = 0.8 mm FOV = 175 mm
Comparing the existence of Fuzzy Ripples across 2D and 3D sequences. This also allows comparisons of the proposed mitigation strategies with DPG.	7	1.01 ms	2D‐EPI,^8^ 3D‐EPI,^6^ WIP 1105^19^	7	Sequence version	GRAPPA 3, axial slab, resolution = 0.8 mm FOV = 175 mm

### 3.1 Experiments to exemplify resolution limits at 3T

Most relevant scan parameters include: SIEMENS Prisma 3T (with XR‐gradient set), 3D‐EPI with isotropic resolutions between 0.8 and 0.53 mm. The full protocol is available here: https://github.com/layerfMRI/Sequence_Github/blob/master/dual‐polarity/3T_inverted_res.pdf Functional activation was induced using a free movie‐watching paradigm, utilizing the same 15‐min movie clips from the 7T HCP study (MOVIE1).

### 3.2 Large FOV data to compare Fuzzy Ripples with edge EPI ghosts

Four participants were scanned to compare Fuzzy Ripples with conventional edge ghosts. A large FOV of 400 mm was used to capture Fuzzy Ripple ghosts independently of the main signal. The main imaging parameters were consistent across experiments: 3D‐EPI, resolution 1 mm isotropic, TE 26 ms, echo spacing 1.02 ms, 7 T with SC72 gradient sets. Comparisons were made with and without: GRAPPA, ramp sampling, a good/bad shim, and dual‐polarity averaging, segmentation factors varied between 2 and 6 to maintain the echo spacing despite varying GRAPPA acceleration. To mitigate contamination of physiological noise with in‐plane segmented acquisition, we only used data (volumes in the time series) that exhibited motion displacement of less than one voxel. Full protocol is available here: https://github.com/layerfMRI/Sequence_Github/blob/master/Terra_protocolls/Fuzzy_ripples/20230424_largeFOV.pdf.

### 3.3 Auditory activation with third‐order shim induced Fuzzy Ripples

To illustrate the impact of Fuzzy Ripples on functional time series, task‐based activation experiments were conducted using the protocols mentioned above. Functional runs followed previous experiments^20^ and consisted of 14 min with alternating 30‐s blocks of rest and auditory stimulation. Sounds were delivered using MRI‐compatible ear buds from Sensimetrics Corporation (www.sens.com), with tones described as “Chipmunks from space”. Sound sample available here: https://youtu.be/TGX_Ulbv9wA?si=VuhMcQj_vDYkibhc. Four experiments were conducted with two participants each participating twice. Protocol parameters included: 0.8 mm resolution, 2D‐multiband sequence from CMRR,^8^ echo spacing 1.0 ms, TE 26 ms, 7T with SC72 gradient sets. Full protocol details are available here: https://github.com/layerfMRI/Sequence_Github/blob/master/Terra_protocolls/Fuzzy_ripples/CMRR_ax_1156_slab.pdf.

Short runs of 50 images were repeated during rest with and without third‐order shims with opposing phase encoding direction. This was done to quantify the amount of geometric distortions in the Supplementary Material.

### 3.4 Alternative mitigation strategies of Fuzzy Ripples

#### 3.4.1 Comparing Fuzzy Ripples across echo spacings and sequences

Six participants were examined to compare Fuzzy Ripples across various echo spacings in popular layer‐fMRI sequences: 3D‐EPI,^6^ CMRR Multiband 2D EPI,^8^ SIEMENS SMS 2D‐EPI, and Dual Polarity GRAPPA (DPG) WIP 1105D.^19^ These experiments were conducted on SIEMENS 7T Terra scanners. Six sessions were carried out on the 7T Terra scanner at NIMH, with two participants additionally scanned on the NMRF Terra. Parameters such as resolution, TR, TE, acceleration, and FOV were matched across echo spacings, with axial slabs covering the temporal lobes using 36 slices. Echo spacings varied between 1 and 1.26 ms. Full protocol details are available here: https://github.com/layerfMRI/Sequence_Github/blob/master/Terra_protocolls/3rd_order_shim/20240528_thirdordershim_siemens.pdf.

#### 3.4.2 Sequence comparison with DPG data

We conducted experiments with seven participants to demonstrate the differences between the proposed dual‐polarity averaging and the previously proposed dual‐polarity GRAPPA (DPG) method.^19^ In the DPG approach, differences between read directions across odd and even EPI lines are corrected for by using a GRAPPA method, where GRAPPA reference data are acquired with two polarities to train separate GRAPPA kernels for odd and even lines. This approach addresses higher‐order phase differences more effectively than conventional EPI corrections, particularly mitigating edge ghosts and B_0_‐related artifacts. However, DPG does not target trajectory errors as shown in Figure 3. Namely, DPG uses an RF‐related reconstruction approach to account for gradient related imperfections which are represented at different scales in k‐space. DPG uses a GRAPPA kernel of finite size to characterize odd‐even mismatches along the entirety of the read line. Thus, sparse odd‐even gradient errors during the read lines cannot be accounted for with DPG. These sparse gradient errors would correspond to lasting but spatiotemporal varying kx shifts (phase mismatch). The read gradient errors that are less sparse might be correctable with DPG to some degree, though.

Due to the available implementation of the DPG method via the SIEMENS WIP package 1105 (VE12U) 2D‐SMS EPI, we compared our 3D‐EPI dual‐polarity averaging implementation with this 2D implementation. We matched echo spacing, resolution, acceleration, TE, and FOV across both sequences. Note that the sequences might use different reconstruction pipelines. Full protocol details are available here: https://github.com/layerfMRI/Sequence_Github/blob/master/Terra_protocolls/Fuzzy_ripples/20230706_segmentationVSGRAPPA.pdf.

### 3.5 Analysis: Image reconstruction, motion correction, dual‐polarity averaging, and activation detection

MRI data were reconstructed on the scanner using MOSAIC.^21^ Dual‐polarity averaging of complex‐valued images was performed offline between odd and even TRs following motion correction to allow analysis of both averaged and raw images.

Motion correction was performed in AFNI (version AFNI_23.2.04) with 3dAllineate. BOLD correction of VASO data was performed in LayNii^22^ (version v2.7.0). Auditory fMRI data were denoised with NORDIC^23^ as optimized and described for this sequence by Knusden et al.^24^ Functional activation analyses were performed with general linear model (GLM) implementation of AFNI's 3dDeconvolve. Layerification was performed in LayNii with the equi‐volume principle in LN2_LAYERS. All analysis scripts are available on Github: https://github.com/layerfMRI/repository. This includes a full script of the preprocessing including complex‐valued motion correction and subsequent dual‐polarity averaging is available on github: https://github.com/layerfMRI/repository/tree/master/Fuzzy_Ripples.

### 3.6 Ethics statement

The scanning procedures at 3T have been approved by the Ethics Review Committee for Psychology and Neuroscience (ERCPN) at Maastricht University, following the principles expressed in the Declaration of Helsinki. 7T results were acquired under the NIH‐IRB (93‐M0170, ClinicalTrials.gov: NCT00001360). We thank Shruti Japee for guidance and support with respect to getting privileges for checking pregnancy tests and IRB.

## 4 RESULTS

### 4.1 Characterization of Fuzzy Ripples compared to other EPI ghosts

**FIGURE 4:**
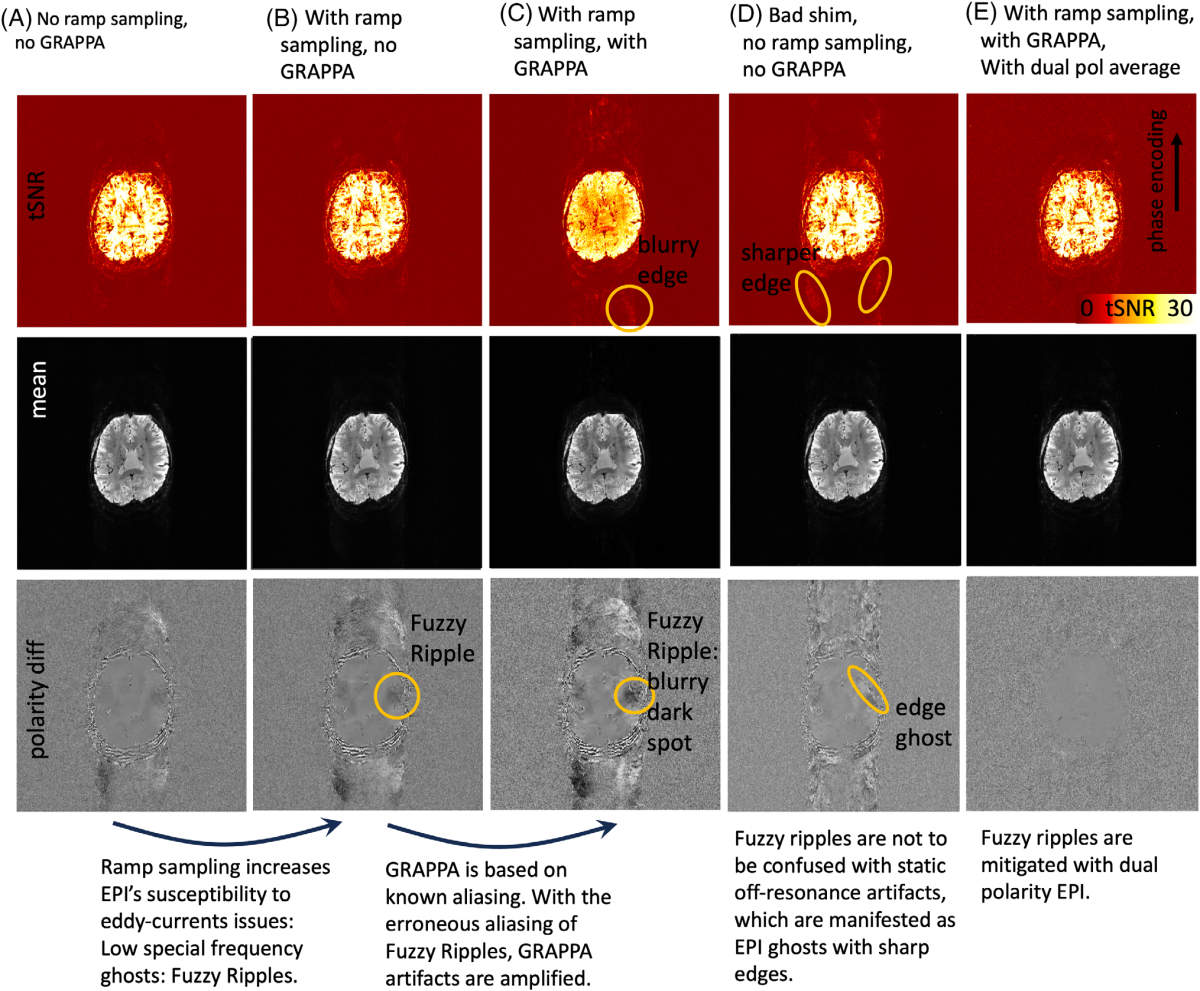
Interaction of Fuzzy Ripples with other common EPI artifacts: GRAPPA ghosts and static off‐resonance ghosts. This figure illustrates EPI acquisitions with different combinations of ramp sampling, poor B_0_ shim, and GRAPPA. The unusually large FOV was purposefully chosen to detect peripheral ghost artifacts. Signal differences between reverse EPI polarity images are shown to emphasize spatial ghost patterns that might be too subtle to observe with conventional image intensity windowing. The read direction in left–right, phase encoding direction is anterior–posterior. (A) Without ramp sampling, imaging data are acquired only during the flat top of the gradient waveform. This minimizes the impact of large gradient errors, resulting in relatively weak Fuzzy Ripples in the EPI images. (B) With ramp sampling enabled, EPI becomes more sensitive to the largest gradient errors, causing Fuzzy Ripples to intensify. These ripples appear as aliasing of low spatial frequencies, with no sharp edges evident in the phase encoding direction. (C) GRAPPA, which relies on a known aliasing pattern, is affected by erroneous Fuzzy Ripple ghosts and thus amplifies their impact. (D) This differs from static off‐resonance effects. For instance, with suboptimal shimming (deliberately altered in this case), the off‐resonance effects do not amplify the low‐spatial frequency Fuzzy Ripples. Instead, they introduce edge ghosts at high spatial frequencies, which differ from Fuzzy Ripples in their appearance. (E) The dual‐polarity averaging approach effectively mitigates both sources of artifacts. The resulting images are nearly artifact‐free. Acquisition parameters of data presented here are mentioned in methods Section 3.3. See Figures S2, S12, and S13 for the reproducibility of these results in participants and on another scanners.

According to the theory of ramp‐sampling EPI, as summarized in Figure 3, Fuzzy Ripples can be described as a result from gradient trajectory imperfections, distinct from off‐resonance‐induced Nyquist ghosts. Figures 4 and S2, S12, S13 highlight the different spatial characteristics of Fuzzy Ripples compared to conventional edge ghosts. It is visible that Fuzzy Ripples are significantly reduced when EPI trajectories do not use ramp sampling (see Figure 4A,D). In such cases, eddy currents are expected to have largely decayed by the time k_x_‐coordinates with high signal power are acquired, leading to edge ghosting being the primary source of artifacts (Figure 4D).

Conversely, when ramp sampling is employed, along with the associated trajectory imperfections closer to the center of k‐space, Fuzzy Ripples become more pronounced (Figure 4C). These aliasing patterns are further exacerbated when GRAPPA is used. However, dual‐polarity averaging effectively mitigates Fuzzy Ripples (Figure 4E).

The findings depicted in Figures 4 and S2, S12, S13 support the idea that Fuzzy Ripples arise from trajectory imperfections linked to eddy currents, rather than from conventional B_0_‐related off‐resonance effects. However, these results do not provide insights into the specific origins of these eddy currents.

### 4.2 Origin of readout‐specific eddy currents

Our investigations, informed by findings from Boulant et al.^17^ on a 11.7T scanner, suggest that inductive coupling between third‐order shim coils and gradient systems can produce significant eddy currents at certain switching rates. Even when EPI Fuzzy Ripple ghosts are relatively mild, respiration‐induced B_0_ fluctuations during fMRI can cause alternating constructive and destructive interference between the main signal and the ghost, potentially reducing fMRI stability and detection sensitivity.

**FIGURE 5:**
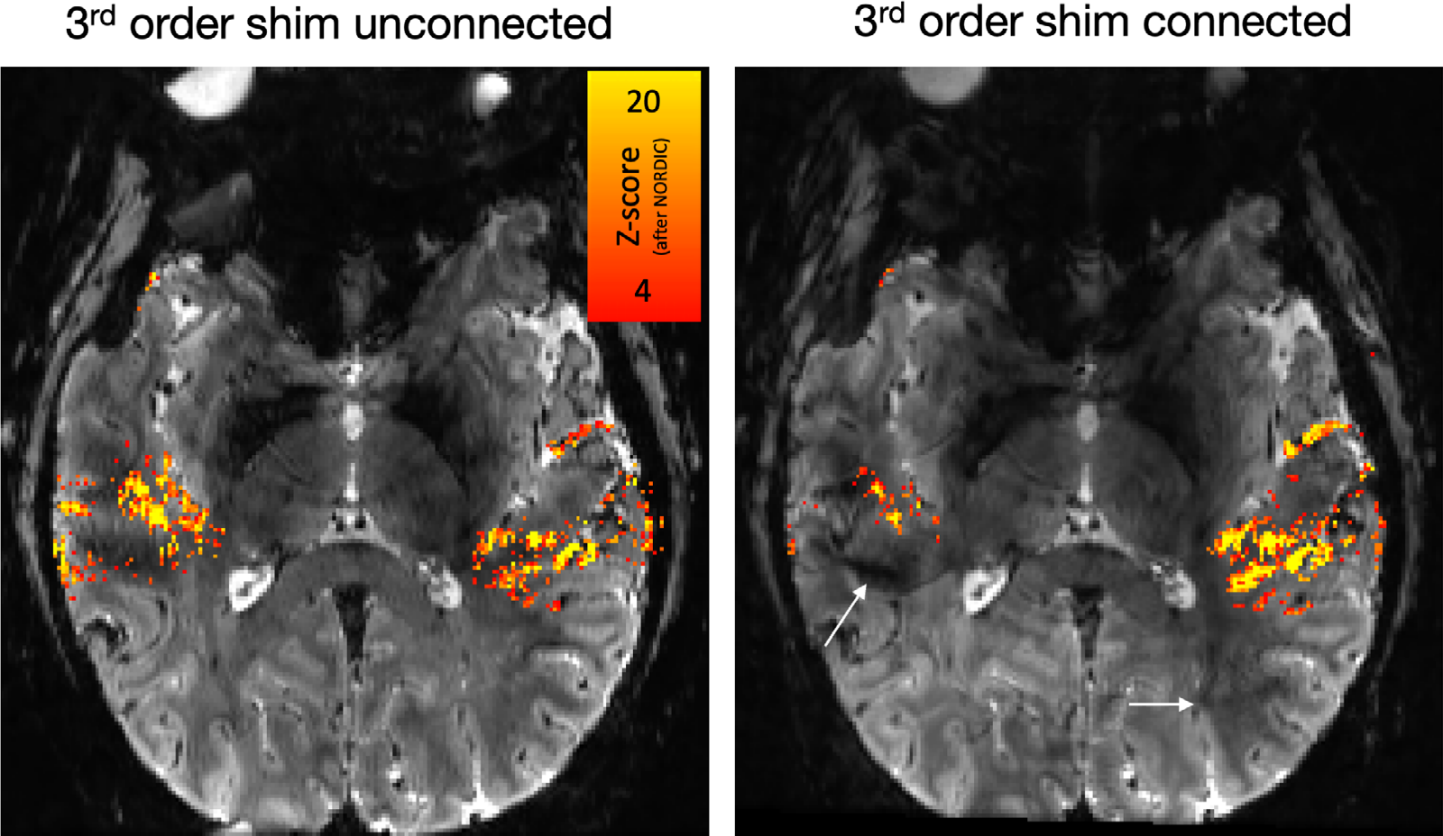
Impact of third‐order shim‐induced Fuzzy Ripples on fMRI activation detectability. This figure demonstrates how Fuzzy Ripples, induced by the third‐order shim, can affect the detectability of fMRI activation. These data refer to 2D‐EPI with block designed auditory activation with NORDIC denoising. When the third‐order shim is connected, the Fuzzy Ripples can be so pronounced that they mask parts of the auditory activation, preventing it from reaching the detection threshold. White arrows indicate areas where the Fuzzy Ripples are more intense with the third‐order shim engaged. These comparisons are performed with single‐polarity EPI acquisitions. Although Fuzzy Ripples are still present when the third‐order shim is disconnected, they are less severe. These residual Fuzzy Ripples that are potentially arising from uncorrected short‐term eddy currents can potentially be reduced with dual‐polarity averaging. Acquisition parameters of data presented here are mentioned in methods Section 3.4. See Figure S3 for a replication of these findings in a different participant.

To explore the impact of third‐order shim coils on high‐resolution fMRI stability, we compared task‐based activation maps obtained with and without the third‐order shims. As shown in Figures 5 and S3, Fuzzy Ripples (indicated by white arrows) are present in areas where there is minimal significant fMRI activity. Unplugging the third‐order shims reduced this artifact. As shown in Figure S9, geometrical distortions are not significantly worse with third‐order shims being disconnected.

### 4.3 Mitigation strategies of Fuzzy Ripples across echo spacings

**FIGURE 6:**
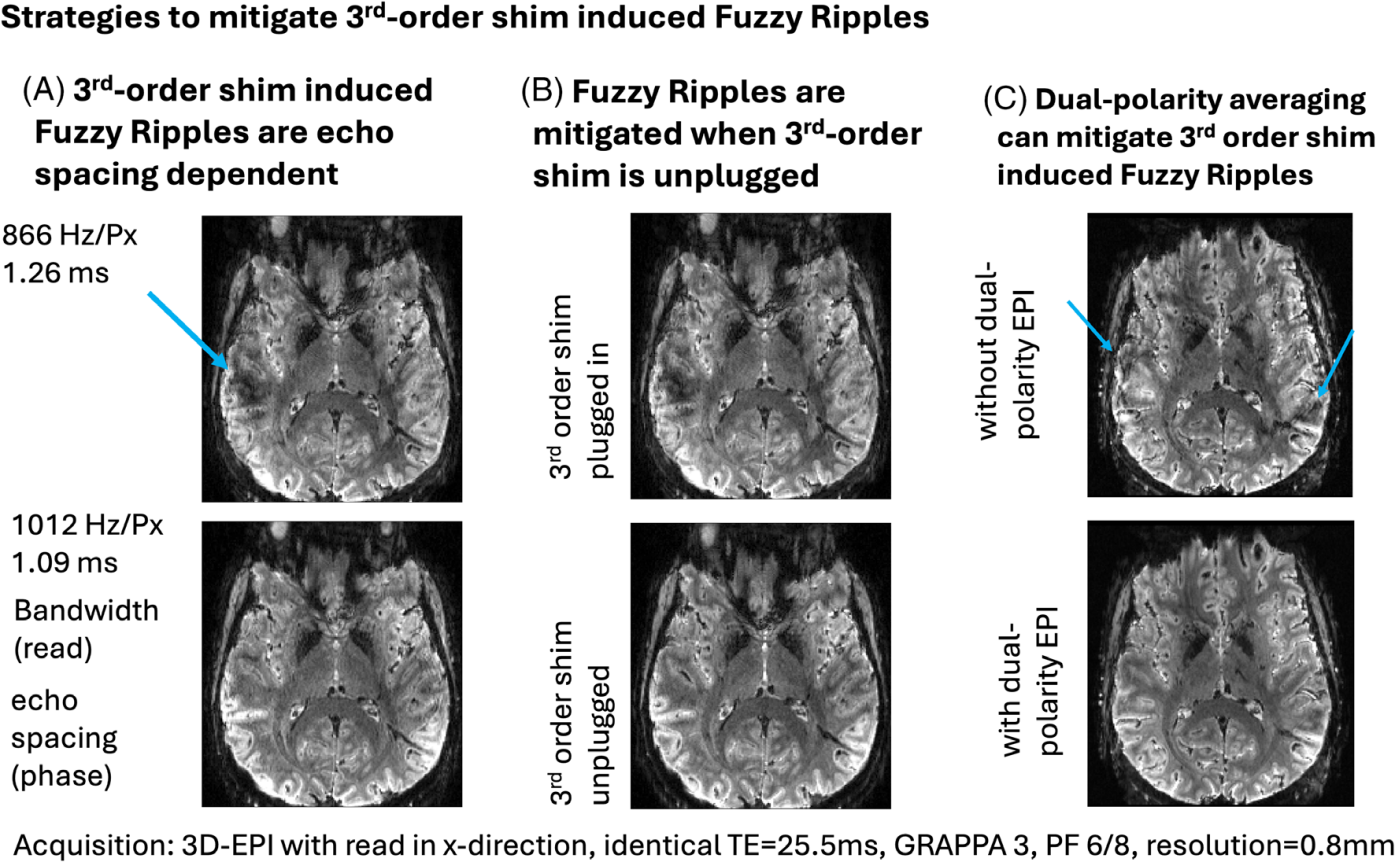
Third‐order shim‐induced Fuzzy Ripples as a function of echo spacing, dual‐polarity averaging, third‐order shim. (A) The Fuzzy Ripple artifact varies with the echo spacing of the EPI readout. Consequently, the strength of this artifact can be reduced by adjusting the readout protocol, although such adjustments may compromise TE and readout efficiency. The adjustment of echo spacing comes along with a different ramp sampling ratio, which can also affect the amount of Fuzzy Ripples. (B) The Fuzzy Ripples induced by the third‐order shim can be mitigated by disconnecting its circuit. Opening this circuit reduces the inductive coupling between the third‐order shim and the gradient and reduces Fuzzy Ripple artifacts. (C) As indicated by Figures 3 and 4, dual‐polarity averaging can counteract the Fuzzy Ripples induced by the third‐order shim. This approach can mitigate Fuzzy Ripples, even for the most problematic echo spacings with the third‐order shim still connected. Though, faint residual Fuzzy Ripples remain. Acquisition parameters of data presented here are mentioned in methods Section 3.5. Figure S4 presents a reproduction of the results shown here.

The results shown in Figures 4 and 5 indicate that Fuzzy Ripples can be mitigated through dual‐polarity averaging and by disabling the third‐order shim, respectively. To evaluate the effectiveness of these mitigation strategies across a broader range of potential fMRI acquisition protocols, we tested them over various EPI echo spacings. The results, presented in Figures 6 and S4, S5, demonstrate the outcomes of these experiments. It is visible that Fuzzy Ripples are most pronounced at echo spacings around 1.26 and 1 ms. The 1.26 ms echo spacing may be related to the fact that the third harmonic of the EPI wave form (1190 Hz) is overlapping with a mechanical resonance of the x/y direction of the SC72 gradient (1100 ± 150 Hz). The 1 ms echo spacing is relatively close to the “forbidden frequencies” (570 ± 20–30 Hz), but safely far enough away from them. This echo spacing is popular in application‐focus layer‐fMRI studies for its time efficiency allowing shortest TRs. The results show that these Fuzzy ripples are mitigated when the third‐order shims are unplugged (Figure 6B). Additionally, even with the third‐order shims enabled, dual‐polarity averaging can reduce the Fuzzy Ripples at the most problematic echo spacing of 1.26 ms (Figure 6C).

### 4.4 Comparison with other dual‐polarity approaches

We evaluated the efficiency of dual‐polarity averaging in comparison to other methods promoted for mitigating EPI ghosting in high‐resolution UHF protocols. These tests were performed with the third‐order shim connected and an echo spacing of 1.01 ms. Such protocols are commonly used in layer‐fMRI because they enable the fastest acquisition times, shortest TE, and matrix sizes of 250–300.

**FIGURE 7:**
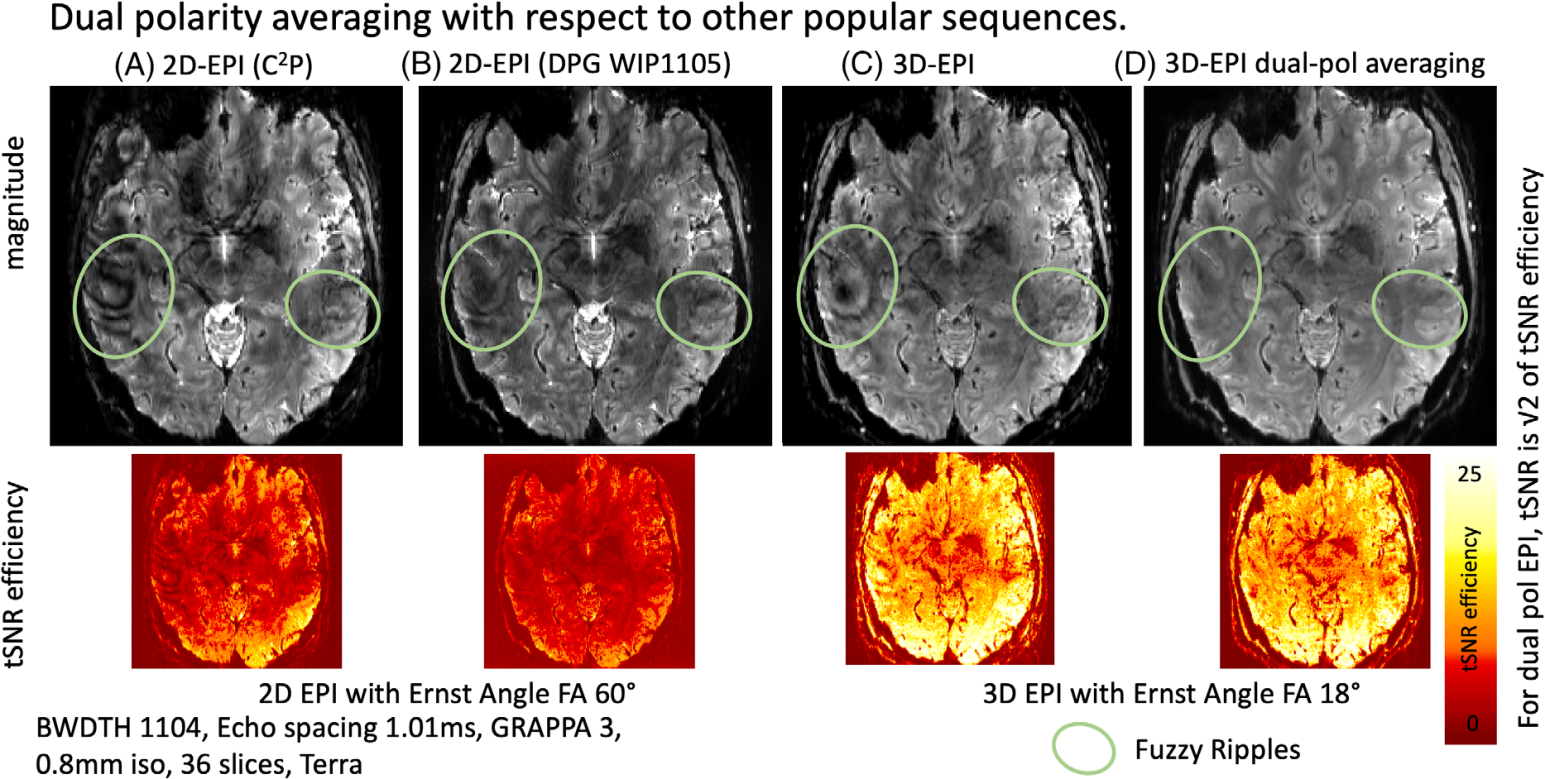
Dual‐polarity averaging with respect to other popular sequences. All sequences are used with the same resolution, echo spacing, and acceleration parameters. (A) This panel shows the CMRR multiband sequence with these protocols, where off‐resonance effects and Fuzzy Ripple artifacts are clearly visible. (B) This panel displays the MGH simultaneous multi‐slice sequence with the option of dual‐polarity GRAPPA. While off‐resonance effects are mitigated, Fuzzy Ripple artifacts, though reduced, remain visible. For more in‐depth investigations of the capability of DPG to account for Fuzzy Ripple artifacts, see Figures S10 and S11. (C) This panel illustrates the same protocols using 3D‐EPI. Due to its different Mz steady‐state behavior, 3D‐EPI inherently has a higher SNR. Additionally, off‐resonance effects are less noticeable, as they are smeared and partially averaged out. However, 3D‐EPI still suffers from Fuzzy Ripples. (D) This panel depicts 3D‐EPI with dual‐polarity averaging. It is visible that Fuzzy Ripples are effectively mitigated. Acquisition parameters of data presented here are mentioned in methods Section 3.5. Figures S5 and S6 presents a reproduction of the results shown here.

Figure 7A shows a 2D‐EPI image using the CMRR multiband sequence, where Fuzzy Ripples (highlighted by green ellipses) are visible. Figure 7B illustrates the use of the dual‐polarity GRAPPA sequence, as conceptualized by Hoge et al.^19^ and distributed for SIEMENS VE as part of the WIP package 1105. This approach reduces some aspects of EPI artifacts very effectively. However, fuzzy dark shading patterns (green ellipses) remain, even with the application of DPG. A kx‐specific dual‐polarity GRAPPA kernel may mitigate these residual Fuzzy Ripples more effectively.^25^ Specifically, the so‐called field‐correcting GRAPPA (FCG) might be able to optimize artifact mitigation without compromises in tSNR.^26^

However, the data DPG as shown here, exhibit a lower temporal SNR (tSNR) compared to single‐polarity data. This reduction in tSNR has previously been hypothesized to be related to the “Sodickson paradox,” which occurs when using larger GRAPPA kernel sizes and less GRAPPA fit regularization. With publicly available reconstruction code that allows for the optimization of GRAPPA parameters, this tSNR reduction might be addressable.

For 3D‐EPI, which we employed in the dual‐polarity averaging approach (Figure 7C,D), we observed that Fuzzy Ripples can be more effectively mitigated.

Note that the panels presented here refer to 2D‐EPI and 3D‐EPI, respectively. While the respective sequences have different T_1_‐steady‐states and tSNR, the in‐plane EPI readout constraints are identical. That is, 3D‐EPI is just added averaging or increased FOV. This means that the occurrence of Fuzzy Ripples across 2D‐ and 3D‐EPI are expected to be identical. Here, we used a combination of methods because of different availability of desired debugging features of widely available sequence baselines (DPG reconstruction, dual polarity, adjustable ramp sampling, and combinability with VASO).

### 4.5 Applications of dual‐polarity EPI in protocols challenged by Fuzzy Ripples

The results presented in Figures 5, 6, 7 demonstrate that the proposed mitigation strategies, including dual‐polarity averaging and disconnecting third‐order shims, can effectively reduce Fuzzy Ripple artifacts. This suggests the possibility of utilizing layer‐fMRI protocols that were previously hindered by Fuzzy Ripples. To illustrate the utility of these mitigation strategies, we applied them to a series of layer‐fMRI protocols that were previously unattainable: (1) higher spatial resolution, (2) faster sampling with aggressive acceleration, and (3) targeting lower brain areas.

Figures S7 and S8 present several examples, including 0.65 mm resolution at 3 T, whole‐brain coverage at 0.64 mm with GRAPPA 2 × 3, and submillimeter fMRI in the cerebellum, among others.

## 5 DISCUSSION

In this project, we investigate a prominent artifact of high‐resolution Cartesian EPI termed as Fuzzy Ripples, that is, low spatial frequency signal shadings.

Based on a meta‐analysis and experiments involving the gradual increase of spatiotemporal resolution across different brain areas (Figures 1 and 2), we propose that the Fuzzy Ripple artifact is not merely a minor issue in conventional layer‐fMRI protocols (0.8 mm, 2–4 s TRs). Instead, Fuzzy Ripples represent a significant source of noise in submillimeter fMRI and pose a greater challenge than thermal noise.

Our empirical studies yielded results that support the hypothesis that Fuzzy Ripples are largely caused by eddy currents in ramp‐sampling EPI, which lead to k_x_‐specific imperfections in gradient trajectories. In the most advanced SIEMENS whole‐body scanners, these eddy currents are largely induced by interactions with the third‐order shim. But at a smaller extent, short‐term eddy currents can also be the cause of Fuzzy Ripples in scanners without third‐order shims. These Fuzzy Ripples are exacerbated in the presence of B_0_ inhomogeneities (as commonly found in lower brain areas), and aggressive GRAPPA accelerations.

We found that the magnitude of Fuzzy Ripples can be reduced using several strategies: (1) disconnecting third‐order shims and (2) employing dual‐polarity averaging. These approaches enable image acquisition that surpasses the current limitations of resolution, sampling rates, and slice prescriptions.

### 5.1 Significance of exceeding current limits in resolution, sampling rates, and flexible slice prescriptions

We have demonstrated that effective strategies can mitigate the Fuzzy Ripple artifact and extend the boundaries of current layer‐fMRI protocols. This advancement has significant implications for studying directional neural information flow within and across brain systems in living humans.

#### 5.1.1 Importance of resolution

While conventional layer‐fMRI resolutions of 0.8 mm isotropic allow researchers to subsample fMRI activity from different laminar neural populations with varying degrees of partial volume effects, these resolutions represent the bare minimum required. Such resolutions do not permit accurate delineation of structural borders without significant partial voluming, nor do they enable the capture of cytoarchitectonically distinct cortical layers with spatial Nyquist sampling. Fuzzy Ripple mitigation strategies allow us to overcome this limit (practical examples shown in Figures S7 and S8). Achieving a 0.46 mm resolution on conventional 7T scanners (Figure S8A) would facilitate the direct observation of laminar activation across the cortical ribbon, addressing some of the criticisms faced by the layer‐fMRI field.^27^ Furthermore, achieving a resolution of 0.53 mm at 3T (Figure S8A) would help disseminate layer‐fMRI beyond the approximately 125 neuroimaging centers worldwide that are equipped with 7T MRI scanners.^21^

#### 5.1.2 Importance of fast sampling

Recently, 0.8 mm layer‐fMRI has been successfully applied with very fast sub‐second acquisition windows.^28^, ^29^, ^30^, ^31^ However, due to the current acquisition constraints related to Fuzzy Ripples, such fast sampling rates have only been possible with small FOVs. While fast whole‐brain imaging is achievable, it is currently limited by Fuzzy Ripple artifacts.^10^ The mitigation methods proposed here could make fast whole‐brain imaging possible with reduced Fuzzy Ripples (practical examples shown in Figures S7 and S8). For example, dual‐polarity averaging has enabled whole‐brain fMRI at 0.6 mm resolutions^5^ and whole‐brain quantitative functional T_1_ mapping.^32^

#### 5.1.3 Importance of lower brain areas

While significant progress has been made in imaging the upper regions of the brain, as evidenced by over 270 published papers, the lower brain areas remain underexplored, hindering the application of whole‐brain layer‐fMRI. Only 3.4% of these publications (www.layerfmri.com/papers) have investigated the lower brain areas, preventing layer‐fMRI from fulfilling its promise of providing a comprehensive whole‐brain functional directional connectome. Numerous important neuroscientific hypotheses involving these lower brain areas remain untested. We are optimistic that the mitigation of Fuzzy Ripple artifacts will pave the way for testing these hypotheses. Some examples include:

- Different layers in the entorhinal cortex, hippocampus, and parahippocampal regions are responsible for memory encoding and retrieval.^33^, ^34^
- Different layers in the fusiform face area (FFA) and parahippocampal place area (PPA) receive feedforward‐feedback input for neural representations of faces and places.^35^, ^36^, ^37^, ^38^
- Different laminar sub‐nuclei of the amygdala are involved in visual perception related to emotional memory versus emotional context.^39^
- Unique lobules of the cerebellum contain sensorimotor digit representations.^40^

Fuzzy Ripple mitigation strategies allow us to overcome these limits (practical examples shown in Figures S7 and S8).

### 5.2 Disadvantages of proposed mitigation strategies

Throughout this study, we explored various independent strategies for mitigating Fuzzy Ripple artifacts, with a focus on dual‐polarity EPI averaging and disconnecting third‐order shims. While these strategies show promise in pushing the boundaries of conventional fMRI protocols, they come with certain compromises.

#### 5.2.1 Unplugging third‐order shim

Unplugging the third‐order shim is a straightforward procedure, supported by vendor‐provided workflows (refer to www.layerfmri.com/3rdordershim for workflows applicable to 7T Terra and 7T Plus scanners). However, this procedure requires a system reboot that takes 10–15 min, which could result in additional costs due to scanner time budgeting. Moreover, some imaging centers are under the false impression that this modification would mean that the scanner would not be in the United States Food and Drug Administration (FDA)‐approved configuration anymore. And there are unfounded institutional worries that unplugging the third‐order shim would have an effect on previously saved imaging protocols. Another drawback of unplugging the third‐order shim is that it may leave fine spatial variations in B_0_ inhomogeneities uncorrected.

#### 5.2.2 Complex‐valued averaging of dual‐polarity EPI

We propose acquiring EPI time series with alternating read polarity, followed by complex‐valued averaging of correspondingimage pairs. When this averaging is conducted for consecutive pairs of images, the resulting averaged data have half the temporal resolution. When this averaging is rather implemented as a sliding window approach, the number of TRs per run is not reduced. However, this sliding window approach may still lead to temporal blurring of signal fluctuations and decouple the fMRI TR from the effective temporal resolution. However, this limitation can be addressed by refraining from pairwise averaging and instead utilizing alternative reconstruction methods:

1. The dual‐polarity approach can be implemented on a calibration‐based, run‐by‐run basis. In this method, phase correction is applied independently to each individual TR without sliding‐window averaging, which avoids temporal smoothing but does not account for temporally varying Fuzzy Ripples. This approach is demonstrated in Steen Moller's implementation within the CMRR MB sequence.^41^
2. Van der Zwaag et al.^13^ introduced the “CP” approach, where the phase difference of each TR pair is estimated separately and applied as a convolution in the projection space, with alternating signs for odd and even TRs. This method prevents temporal smoothing but requires more substantial changes in image reconstruction.
3. Alternatively, one could forgo pairwise averaging and instead account for Fuzzy Ripples in alternating EPI acquisitions through a regression approach during functional activation analysis (as the artifacts alternate between odd/even TRs). This strategy is only feasible if the stimulation and task design are not locked to odd/even TRs.
4. Alternatively, the measured trajectory imperfections could be used in a 1D non‐Cartesian reconstruction model, which could solve the artifact without the need for dual‐polarity calibration.

#### 5.2.3 Adjusting echo spacing

During our experiments that were aimed at characterizing the spatial features of Fuzzy Ripples relative to other artifacts, we identified additional scan parameters that may warrant further investigation as potential mitigation strategies for Fuzzy Ripples. These include avoiding ramp sampling (Figures 4 and S2, S12, S13), avoiding GRAPPA (Figures 4 and S2, S12, S13), and adjusting echo spacing (Figures 6 and S4, S5). While ramp sampling and GRAPPA are essential components of modern, efficient fMRI protocols, further exploration of echo spacing adjustments may be justified to quantify its effectiveness as a Fuzzy Ripple mitigation strategy.

Fine‐tuning echo spacing is a common optimization step performed during the piloting phase of any layer‐fMRI protocol. This process is often undertaken to avoid overlap with mechanical resonances of the main EPI frequency or potential sidebands from non‐sinusoidal gradient pulses. This study underscores the importance of such optimizations and suggests that echo spacing should also be optimized specifically for Fuzzy Ripple artifacts. However, this approach is constrained by the desired TEs. For matrix sizes greater than 200, the tradeoff in TE could be as large as 3–12 ms, potentially leading to increased signal decay, blurring, and spatial distortions.

#### 5.2.4 Potential reconstruction with measured trajectories

In this study, we used the SKOPE field camera in initial phantom experiments to obtain a well‐defined research question and to conceptualize the dimensionality of the problem we wanted to address. These measured k‐space trajectories, however, might also have utility in potential future reconstruction pipeline in application‐focused fMRI data themselves. For example, in the future, trajectory mapping (e.g., with SKOPE) might become more widely available to application‐focused neuroscience imaging centers and field‐specific nonlinear 3D‐EPI reconstruction methods will become efficient enough for real time efficiency. Then, Fuzzy Ripples can also be solved in a forward reconstruction model without the need of the strategies discussed here.

#### 5.2.5 Potential filtering with UNFOLD

Outside of the field of layer‐fMRI, EPI artifacts in cardiac imaging have been proposed to be accounted for by Unaliasing by Fourier‐Encoding the Overlaps Using the Temporal Dimension (UNFOLD).^42^ This artifact mitigation approach has been developed to address EPI problems, when the underlying signals vary during the acquisition of a single volume acquisition. It has been further developed for use in self‐references DPG imaging.^43^ Making it comparatively similar to virtual “interleaved flyback” approach.^15^ The main commonly discussed disadvantage of the application of UNFOLD is the potential of limiting noise amplification when using high acceleration factors. In the case of thermal noise limited high‐resolution fMRI, the supralinear noise amplifications with acceleration (g‐factor >1) outweighs the higher temporal resolution and image and sample counts.

An additional concern with UNFOLD‐based fMRI reconstruction has been discussed in the literature.^44^ Namely, a transient head position change at a single imaging time point may be translated to artifacts in multiple imaging time points through temporal‐domain data filtering.

Future work of combining UNFOLD into one of the commonly used sequences in the field of layer‐fMRI will be able to quantify the utility of this approach within the thermal noise limited regime of sub‐millimeter resolution fMRI.

## 6 CONCLUSIONS

In this study, we have characterized a significant EPI artifact termed as Fuzzy Ripples, which poses a substantial limitation for laminar imaging. This low spatial resolution EPI ghosting artifact is caused by trajectory imperfections in ramp sampling EPI, restricting achievable spatial resolution, sampling efficiency, and flexibility of FOV prescriptions. Based on the insights of the origin of this artifact from this study, we proposed several mitigation strategies, including dual‐polarity EPI and disconnecting third‐order shims. Our findings indicate that these strategies can effectively mitigate Fuzzy Ripple artifacts, thereby extending the capabilities of layer‐fMRI acquisition protocols.

## FUNDING INFORMATION

Renzo Huber is supported by the NIH Intramural Program of NIMH/NINDS (#ZIC MH002884). Benedikt Poser is partially funded by the NWO VIDI grant 16.Vidi.178.052. Drs. Poser, Ma, Stirnberg, Stöcker, Boulant received financial support from the European Union Horizon 2020 Research and Innovation program under grant agreement 885876 (AROMA). Andrew Persichetti is supported by the NIH Intramural Program of NIMH (ZIA‐MH‐002920‐09) and a K99 award from the National Eye Institute (K99EY034169–01). The acquisition of whole brain data shown in Figure S8B was supported by the BRAIN Initiative (NIH grants R0‐MH111444 and U01‐EB025162), and NIH R44‐MH129278. The brain data in Figure S1A were acquired with funds from the NWO VENI project 016.Veni.198.032. The data acquisition for brain data shown in Figures 1B and S8A was kindly provided as “development time” by the UM faculty of Psychology and Neuroscience. Measurement data in Figure 3A were acquired with the kind support of the companies Scannexus and Skope.

## CONFLICT OF INTEREST STATEMENT

Omer Faruk Gulban is an employee of Brain Innovation (Maastricht, NL). David Feinberg owns the company Advanced MRI technologies, LLC. The work presented here may be partly specific to industrial design choices of SIEMENS Healthineers' UHF scanners. This vendor is used in 83% of all human layer‐fMRI papers (source: www.layerfmri.com/papers).

## Supporting information


**Figure S1.** Depicts how Fuzzy ripples are keeping us from achieving higher resolutions, shorter TRs, and lower brain areas.
**Figure S2, S12, S13.** Shows how Fuzzy Ripples are different from other EPI ghostings.
**Figure S3.** Shows how Fuzzy Ripples compromise task activation maps.
**Figures S4‐S6.** Show that the results are generalizable across scanners and participants.
**Figures S7‐S8.** Show applications of the proposed method for higher resolution, lower brain areas, and faster sampling.
**Figures S9.** Depicts the effect of third order shims on Fuzzy Ripples.
**Figures S10‐S11.** Depict the effect of DPG on Fuzzy Ripples.

## Data Availability

Scanning protocols with complete sets of sequence parameters are available here: https://github.com/layerfMRI/Sequence_Github/tree/master/Terra_protocolls/. Data on Temporal resolution are available on Openneuro: https://doi.org/10.18112/openneuro.ds003216.v3.0.11. The LayNii software used here is available on Zenodo: https://zenodo.org/doi/10.5281/zenodo.3514297. A full script of the preprocessing including complex‐valued motion correction and subsequent dual‐polarity averaging is available on github: https://github.com/layerfMRI/repository/tree/master/Fuzzy_Ripples. Raw data of third‐order shim across echo spacings are here: https://layerfmri.page.link/3rdShim_data. Raw data of B_0_‐related distortions with and without third‐order shims are available here: https://doi.org/10.5281/zenodo.13323821. Additional reproducibility data large FOV experiments about the distinction of Fuzzy Ripple artifacts compared to other forms of odd‐even artifacts are available here: https://doi.org/10.5281/zenodo.13323821. 3D‐EPI sequences (incl. VASO) with optional dual‐polarity EPI and on‐scanner reconstruction of coil‐specific dual‐polarity averaging can be accessed via the Siemens C2P exchange platform: For European IP addresses https://webclient.eu.api.teamplay.siemens‐healthineers.com/c2p and for US IP addresses https://webclient.us.api.teamplay.siemens‐healthineers.com/c2p (v2.0 at time of submission). Search for 3D‐EPI (by Stirnberg and Huber) provided by DZNE. The auditory data and the data across sequences and across bandwidth are available here: https://zenodo.org/records/13326459. Raw data of SKOPE trajectories and the first‐order fits of linear gradients are available here: https://doi.org/10.5281/zenodo.13323821.

## References

[1] Ahn CB , Cho ZH . A new phase correction method in NMR imaging based on autocorrelation and histogram analysis. IEEE Trans Med Imaging. 1987;6:32‐36. doi:10.1109/TMI.1987.4307795 18230424

[2] Feiweier T , inventor; Siemens Aktiengesellschaft, assignee . Magnetic resonance method and apparatus to determine phase correction parameters. US Patent 8,497,681. July 30, 2013.

[3] Heid O . Method for the phase correction of nuclear magnetic resonance signals, Patent: US006043651A. 2000.

[4] Deshmane A , Gulani V , Griswold MA , Seiberlich N . Parallel MR imaging. Magn Reson Imaging. 2012;36:55‐72. doi:10.1002/jmri.23639 PMC4459721 22696125

[5] Feinberg DA , Beckett AJS , Vu AT , et al. Next‐generation MRI scanner designed for ultra‐high‐resolution human brain imaging at 7 tesla. Nat Methods. 2023;20:2048‐2057. doi:10.1038/s41592-023-02068-7 38012321 PMC10703687

[6] Stirnberg R , Stöcker T . Segmented K‐space blipped‐controlled aliasing in parallel imaging (skipped‐CAIPI) for high spatiotemporal resolution Echo planar imaging. Magn Reson Med. 2020;85:1540‐1551. doi:10.1101/2020.06.08.140699 32936488

[7] Huber L , Finn ES , Chai Y , et al. Layer‐dependent functional connectivity methods. Prog Neurobiol. 2021;207:101835. doi:10.1016/j.pneurobio.2020.101835 32512115 PMC11800141

[8] Moeller S , Yacoub E , Olman CA , et al. Multiband multislice GE‐EPI at 7 tesla, with 16‐fold acceleration using partial parallel imaging with application to high spatial and temporal whole‐brain FMRI. Magn Reson Med. 2010;63:1144‐1153. doi:10.1002/mrm.22361 20432285 PMC2906244

[9] Graedel NN , Kasper L , Engel M , et al. Feasibility of spiral fMRI based on an LTI gradient model. Neuroimage. 2021;245:118674. doi:10.1016/j.neuroimage.2021.118674 34718138

[10] Koiso K , Müller AK , Akamatsu K , et al. Acquisition and processing methods of whole‐brain layer‐fMRI VASO and BOLD: the Kenshu dataset. Aperture Neuro. 2023;34: 1–22. doi:10.1101/2022.08.19.504502 PMC11981596 40206493

[11] Bernstein MA , King KF , Zhou XJ . Handbook of MRI Pulse Sequences. Elsevier Academic Press; 2004.

[12] Feinberg DA , Oshio K . Phase errors in multi‐shot echo planar imaging. Magn Reson Med. 1994;32:535‐539. 7997122 10.1002/mrm.1910320418

[13] van der Zwaag W , Marques JP , Lei H , Just N , Kober T , Gruetter R . Minimization of Nyquist ghosting for echo‐planar imaging at ultra‐high fields based on a “negative readout gradient” strategy. J Magn Reson Imaging. 2009;30 November 2009:1171‐1178. doi:10.1002/jmri.21951 19856451

[14] Poser BA , Barth M , Goa P‐E , Deng W , Stenger VA . Single‐shot Echo‐planar imaging with Nyquist ghost compensation: interleaved dual Echo with acceleration (IDEA) Echo‐planar imaging (EPI). Magn Reson Med. 2013;69:37‐47. doi:10.1002/mrm.24222 22411762 PMC3378781

[15] Stirnberg R , Deistung A , Reichenbach JR , Breteler MMB , Stöcker T . Rapid submillimeter QSM and R_2_* mapping using interleaved multishot 3D‐EPI at 7 and 3 tesla. Magn Reson Medmrm. 2024;92:2294‐2311. doi:10.1002/mrm.30216 38988040

[16] Hu X , Le TH . Artifact reduction in EPI with phase‐encoded reference scan. Magn Reson Med. 1996;36:166‐171. doi:10.1002/mrm.1910360126 8795036

[17] Boulant N , Le Ster C , Amadon A , et al. The possible influence of third‐order shim coils on gradient–magnet interactions: an inter‐field and inter‐site study. Magn Reson Mater Phys Biol Med. 2024;37:169‐183. doi:10.1007/s10334-023-01138-3 PMC10995016 38197908

[18] Poser BA , Koopmans PJ , Witzel T , Wald LL , Barth M . Three dimensional echo‐planar imaging at 7 tesla. Neuroimage. 2010;51:261‐266. doi:10.1016/j.neuroimage.2010.01.108 20139009 PMC2853246

[19] Hoge WS , Polimeni JR . Dual‐polarity GRAPPA for simultaneous reconstruction and ghost correction of Echo planar imaging data. Magn Reson Med. 2016;76:32‐44. doi:10.1002/mrm.25839 26208304 PMC4758917

[20] Faes LK , De Martino F , Huber L . Cerebral blood volume sensitive layer‐fMRI in the human auditory cortex at 7T: challenges and capabilities. PLoS One. 2023;18:e0280855. doi:10.1371/journal.pone.0280855 36758009 PMC9910709

[21] Huber L , Kronbichler L , Stirnberg R , et al. Evaluating the capabilities and challenges of layer‐fMRI VASO at 3T. Aperture Neuro. 2023;3: 1–17. doi:10.52294/001c.85117 PMC11845223 39991189

[22] Huber L , Poser BA , Bandettini PA , et al. LAYNII: a software suite for layer‐fMRI. Neuroimage. 2021;237:118091. doi:10.1016/j.neuroimage.2021.118091 33991698 PMC7615890

[23] Vizioli L , Moeller S , Dowdle L , et al. Lowering the thermal noise barrier in functional brain mapping with magnetic resonance imaging. Nat Commun. 2021;12:5181. doi:10.1038/s41467-021-25431-8 34462435 PMC8405721

[24] Knudsen L , Vizioli L , Martino FD , et al. NORDIC denoising on VASO data. Front Neurosci. 2025; 18:1499762. doi:10.3389/fnins.2024.149976218 39834697 PMC11743533

[25] Wang N , Abraham D , Kerr AB , et al. Field‐Correcting GRAPPA (FCG) For Improved Mitigation Of Even‐Odd And Field‐Related Artifacts In EPI. ISMRM; 2024; 1256.

[26] Wang N , Abraham D , Wu H , et al. Field‐Correcting GRAPPA (FCG): a generalizable technique to correct spatiotemporal varying odd‐even phase errors in EPI, SMS‐EPI and 3D‐EPI, ISMRM. 2025; 7572.

[27] Weldon KB , Olman CA . Forging a path to mesoscopic imaging success with ultra‐high field functional magnetic resonance imaging. Phil Trans R Soc Lond B Biol Sci. 2020;376:20200040. doi:10.1098/rstb.2020.0040 33190599 PMC7741029

[28] Dresbach S , Huber R , Gulban OF , Goebel R . Layer‐fMRI VASO with short stimuli and event‐related designs at 7T. Neuroimage. 2023;279:120293. doi:10.1016/j.neuroimage.2023.120293 37562717

[29] Dresbach S , Huber R , Gulban OF , et al. Characterisation of laminar and vascular spatiotemporal dynamics of CBV and BOLD signals using VASO and ME‐GRE at 7T in humans. Imaging Neurosci. 2024; 2:1–12. doi:10.1101/2024.01.25.576050 PMC12290604 40800482

[30] Gomez DEP , Polimeni JR , Lewis LD . The temporal specificity of BOLD fMRI is systematically related to anatomical and vascular features of the human brain. Imaging Neurosci. 2024;2:1‐18. doi:10.1101/2024.02.01.578428 PMC12315723 40800285

[31] Vizioli L , Yacoub E , Lewis LD . How pushing the spatiotemporal resolution of fMRI can advance neuroscience. Prog Neurobiol. 2021;207:102184. doi:10.1016/j.pneurobio.2021.102184 34767874

[32] Kan C(K) , Stirnberg R , Montequin M , et al. T1234: a distortion‐matched structural scan solution to misregistration of high resolution fMRI data. Magn Reson Med. 2025;94:713‐723. doi:10.1002/mrm.30480 10.1002/mrm.30480 PMC12137766 40079433

[33] Zhang K , Chen L , Li Y , et al. Differential laminar activation dissociates encoding and retrieval in the human medial and lateral entorhinal cortex. J Neurosci. 2023;43:2874‐2884. doi:10.1523/JNEUROSCI.1488-22.2023 36948584 PMC10124959

[34] Pfaffenrot V , Bouyeure A , Gomes CA , Kashyap S , Axmacher N , Norris DG . Characterizing BOLD activation patterns in the human hippocampus with laminar fMRI. BioRxiv. 2024. doi:10.1101/2024.07.04.60206 PMC12319889 40800946

[35] Carricarte T , Iamshchinina P , Trampel R , Chaimow D , Weiskopf N , Cichy RM . Laminar dissociation of feedforward and feedback signals in high‐level ventral visual cortex during imagery and perception. PsyArXiv. 2023; 27:110229. doi:10.31234/osf.io/7zcp8 PMC11246059 39006482

[36] Persichetti AS , Audrain S , Huber L , Martin A . Investigating layer‐specific responses to mental imagery and perception in ventral occipital temporal cortex. VSS; 2023.

[37] Dowdle LT , Ghose G , Moeller S , Ugurbil K , Yacoub E , Vizioli L . Task demands differentiate regional depth‐dependent activity profiles within the ventral visual pathway (preprint). BioRxiv. 2022. doi:10.1101/2022.12.03.518973

[38] Koiso K , Akamatsu K , Huber L , Miyawaki Y , OHBM . Laminar‐level object information representation in higher visual areas revealed by VASO layer fMRI, #1788. Proceedings of OHBM Publisher is Organization of Human Brain Mapping. 2023: 1788.

[39] Geissberger N , Tik M , Sladky R , et al. Reproducibility of amygdala activation in facial emotion processing at 7T. Neuroimage. 2020;211:116585. doi:10.1016/j.neuroimage.2020.116585 31996330

[40] Brouwer EJP , Priovoulos N , Hashimoto J , Van Der Zwaag W . Proprioceptive engagement of the human cerebellum studied with 7T‐fMRI. Imaging Neurosci. 2024;2:1‐12. doi:10.1162/imag_a_00268 PMC12290657 40800446

[41] Huber L , Stirnberg R , Morgan AT , et al. Fuzzy ripple artifacts in layer‐fMRI EPI: Towards better layer‐fMRI data with Dual‐polarity readouts, in: OHBM Submitted. 2023.

[42] Madore B , Glover GH , Pelc NJ . Unaliasing by Fourier‐encoding the overlaps using the temporal dimension (UNFOLD), applied to cardiac imaging and fMRI. Magn Reson Med. 1999;42:813‐828. 10542340 10.1002/(sici)1522-2594(199911)42:5<813::aid-mrm1>3.0.co;2-s

[43] Hoge S , Tan H , Kraft O . Improved Self‐Referenced Parallel MRI Imaging in EPI by Using UNFOLD to Remove Nyquist Ghosts. Proceeding ISMRM 17th Scientific Meeting; ISMRM; 2009:2720.

[44] Chang H‐C , Gaur P , Chou Y , Chu M‐L , Chen N . Interleaved EPI based fMRI improved by multiplexed sensitivity encoding (MUSE) and simultaneous multi‐band imaging. PLoS One. 2014;9:e116378. doi:10.1371/journal.pone.0116378 25549271 PMC4280209

