# Supplementary material for: Short‐term gradient imperfections in high‐resolution EPI lead to Fuzzy Ripple artifacts
Source: Magn Reson Med. 2025 Apr 2;94(2):571–87. doi: 10.1002/mrm.30489 (PMC12137764; doi:10.1002/mrm.30489)
Supplement: Supplementary file 1 — Figure S1. Depicts how Fuzzy ripples are keeping us from achieving higher resolutions, shorter TRs, and lower brain areas. Figure S2, S12, S13. Shows how Fuzzy Ripples are different from other EPI ghostings. Figure S3. Shows how Fuzzy Ripples compromise task activation maps. Figures S4‐S6. Show that the results are generalizable across scanners and participants. Figures S7‐S8. Show applications of the proposed method for higher resolution, lower brain areas, and faster sampling. Figures S9. Depicts the effect of third order shims on Fuzzy Ripples. Figures S10‐S11. Depict the effect of DPG on Fuzzy Ripples. [file MRM-94-571-s001.pdf]

Supplementary information on Fuzzy Ripples:

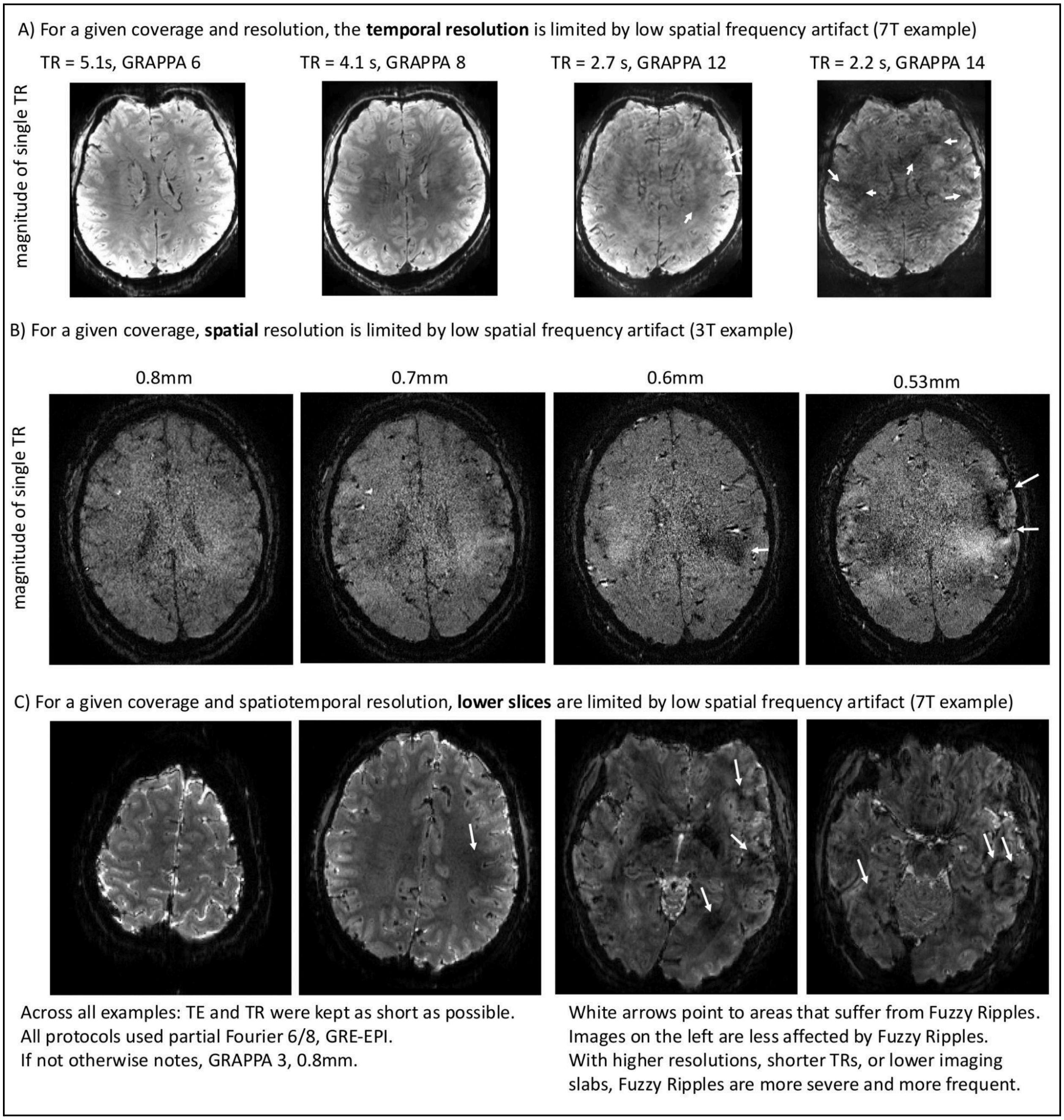

Fig S1: Extension to Fig.1 Fuzzy Ripples are the reason why layer-fMRI is confined to conventional protocols.
Fuzzy Ripples are the primary reason why layer-fMRI is restricted to conventional protocols. Standard layer-fMRI protocols are generally limited to 0.8mm resolution with TRs of several seconds, focusing on upper cortical brain areas. These limitations cannot be surpassed because, with more ambitious acquisition protocols. Fuzzy Ripple artifacts become too strong and too frequent. The example shown here exemplifies issues of pushing resolutions beyond 0.8mm. Panel B is the same as in Fig. 1.
Images from Panels A are taken from Koiso 2023 and Huber 2023, respectively. They refer to 3D-EPI readouts with planar EPI trajectories.
The Skope date shown in Fig. 3 are referring to the trajectories of the imaging data in panel A.

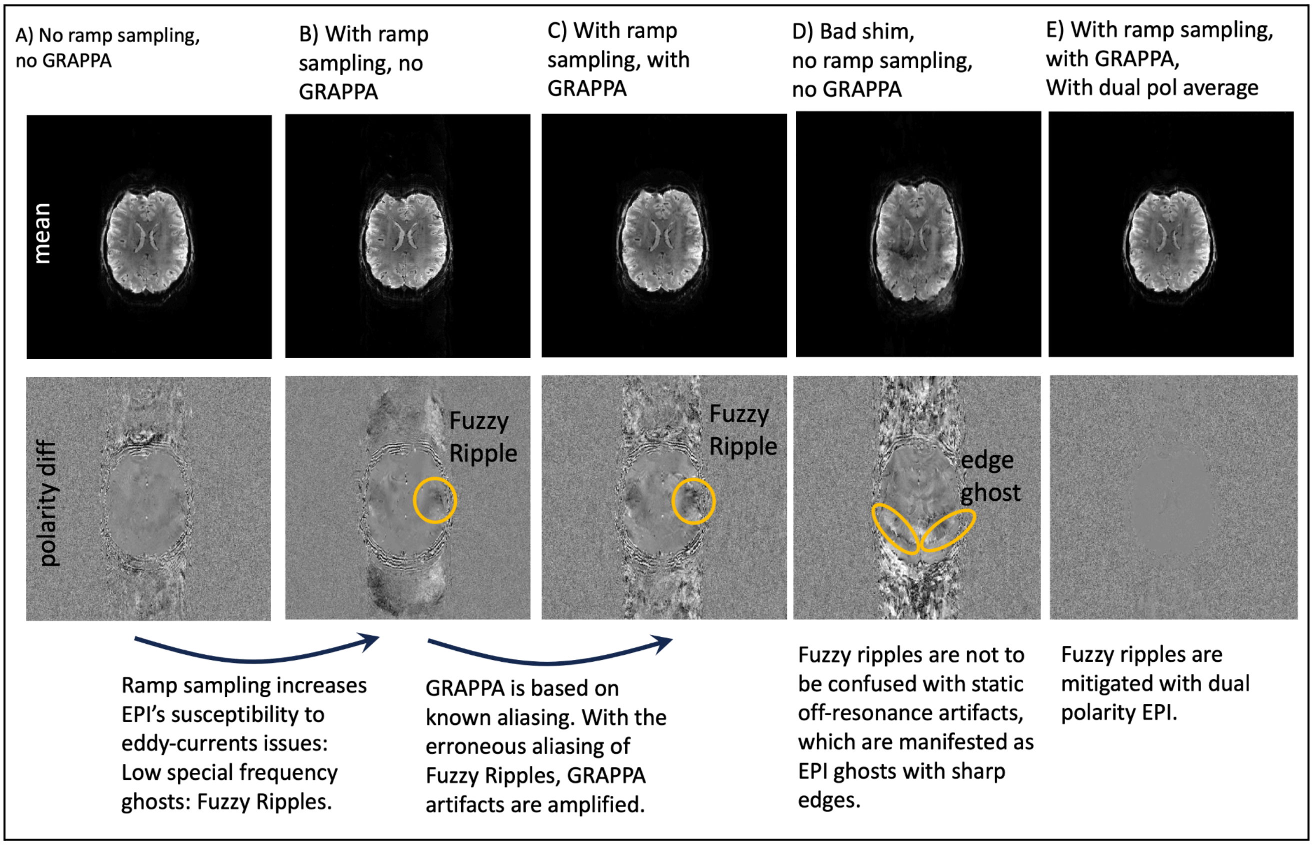

Figure S2: reproduced results of Fig. 4.: GRAPPA ghosts and static off-resonance ghosts.
A series of EPI acquisitions with various combinations of ramp sampling, bad B0 shim and GRAPPA are shown. The FOV purposefully chosen to be unconventionally large, to allow detections of the ghosts in the periphery. The signal difference between reverse EPI polarity images is shown to highlight the spatial ghost pattern that might be too weak to see with conventional image intensity windowing. The read direction in left-right, phase encoding direction is anterior-posterior.

A). *When EPI is done without ramp-sampling, imaging data are solely obtained during the flat top, which eliminates some parts of the largest gradient errors and, thus, the resulting EPI images only show relatively weak Fuzzy Ripples.*

B). *When ramp sampling is turned on, EPI becomes additionally sensitive to the largest peaks in gradient errors. Thus the Fuzzy Ripples become stronger. The Fuzzy Ripples manifest as aliasing of low spatial frequencies, as expected. Note that there are no sharp edges in the phase encoding direction.*

C). *Since, GRAPPA relies on a known aliasing pattern, which is contaminated with erroneous Fuzzy Ripples, it amplifies the effect.*

D). *This is different from static off-resonance effects. For example, in presence of suboptimal shimming (here purposefully altered), it does not amplify the low-spatial frequency fuzzy ripples. Instead, such settings add another source of artifact, namely the edge ghosts at high-spatial frequencies. These sharp borders look different from Fuzzy Ripples. Note that the edge is only sharp along the phase encoding direction. The Fuzzy Ripples that are also amplified with bad shim are smooth in the read direction,*

E). *The dual polarity approach can account for both of these sources of artifacts. The resulting images end up almost perfectly flat.*

Acquisition parameters of data presented here are mentioned in methods section 3.3.
For additional variables investigated in this fashion, see Fig. S12-S13.

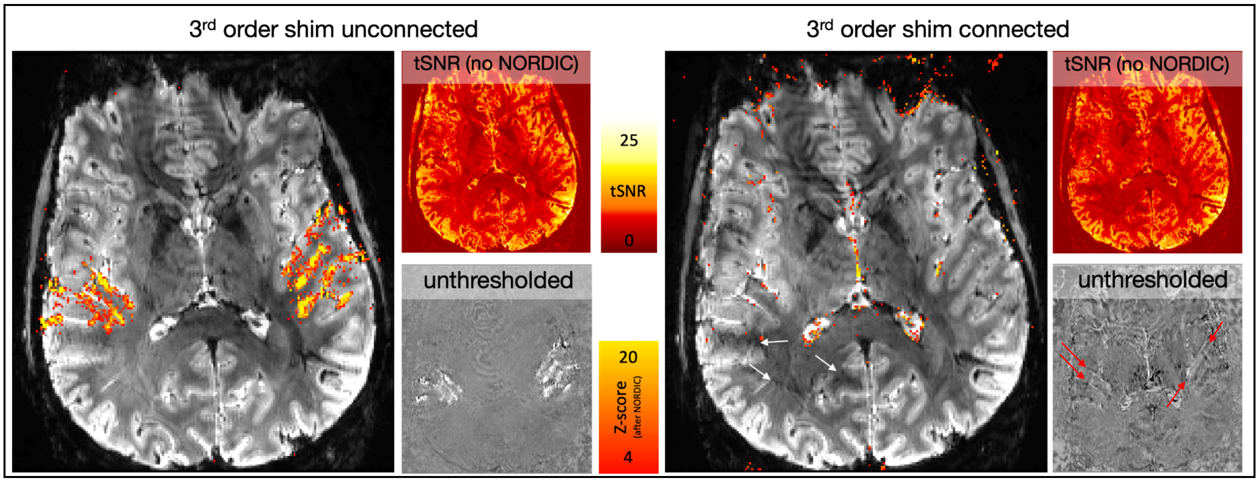

Figure S3: reproduced results of Fig. 5. How 3rd order shim induced Fuzzy Ripples affects fMRI activation detectability.

When the third order shim is connected, Fuzzy Ripples can be so strong that parts of auditory activation do not exceed the detection threshold.

White arrows point to Fuzzy Ripples that are stronger when scanning with the 3rd order shim connected. Fuzzy Ripples are still somewhat present without the 3rd order shim, they are weaker though.

Red arrows highlight activated brain areas. They are visible in unthresholded activation maps. However, they are below the detection threshold.

Acquisition parameters of data presented here are mentioned in methods section 3.4.

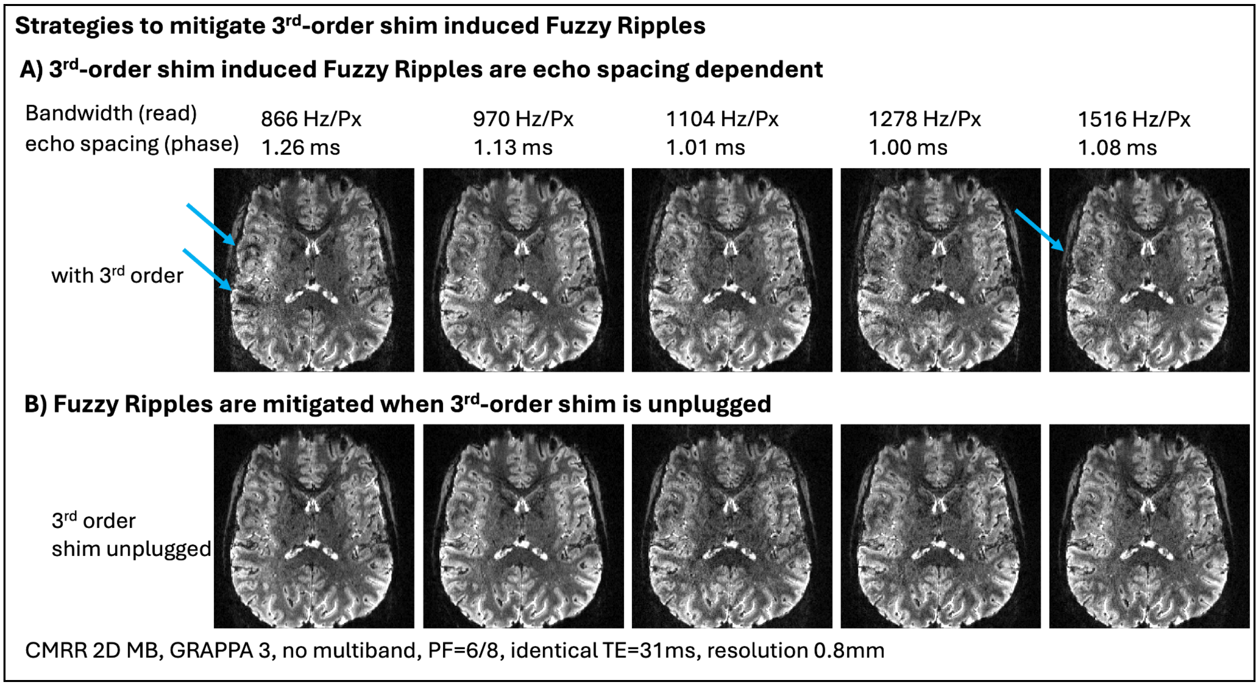

Fig. S4: reproduced results of Fig. 6 on a different scanner with a different participant using a different EPI sequence: 3rd-order shim induced Fuzzy Ripples as a function of echo spacing, cable connected, dual polarity averaging.

A.) The Fuzzy Ripple artifact is dependent on the echo spacing of the EPI readout. Thus, the artifact strength can be mitigated by protocol adjustments of the readout, which might come along with compromises of TE and readout efficiency.

B.) 3rd-order shim induces Fuzzy Ripples can be mitigated by means of unplugging its circuit. Breaking this circuit reduces the inductive coupling of the 3rd-order shim with the gradient.

Dual Polarity averaging results shown in Fig. 6C could not be reproduced with the CMRR sequence. Lacking access to the sequence source code, we could not make the required modifications to the sequence. For pilot tests of dual polarity averaging of this sequence in a phantom see (Huber 2023).

Acquisition parameters of data presented here are mentioned in methods section 3.5.

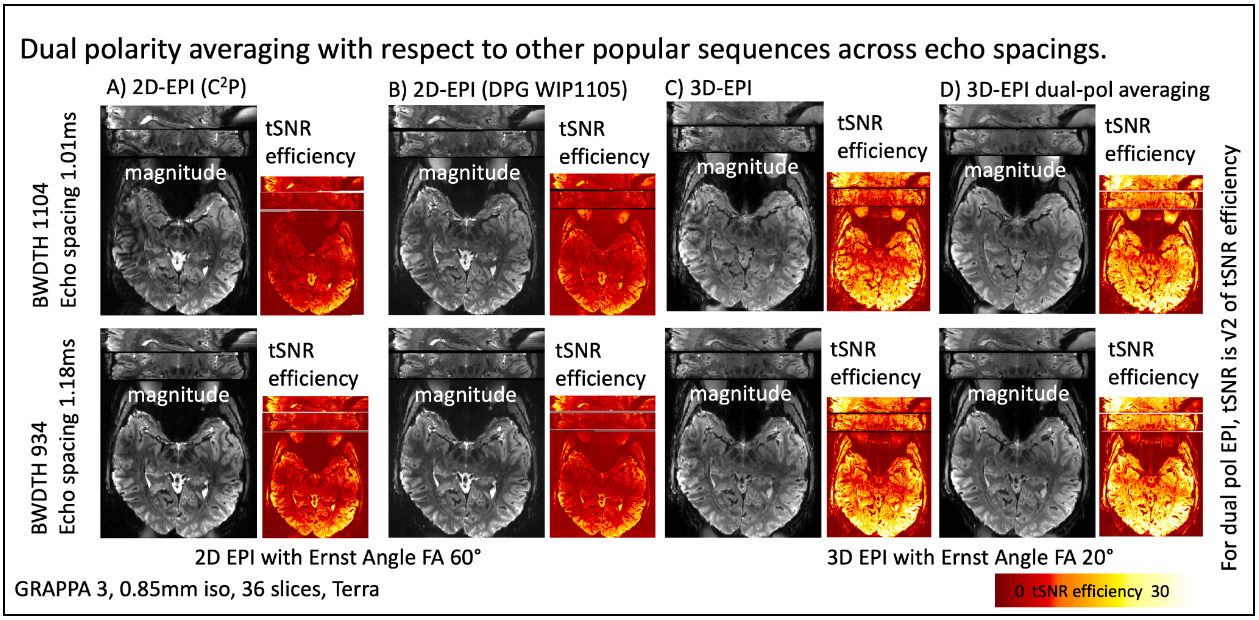

Fig. S5: reproduced results of Fig. 7 on a different scanner with a different participant with different echo spacings.

All sequences are used with the same resolution, and acceleration parameters. Echo spacing is constant for results in each row, respectively.

A) depicts the CMRR multiband sequence with these protocols. Fuzzy Ripple artifacts are visible with different strengths across echo spacings.

B) depicts the MGH simultaneous multi slice sequence of this protocol with its option of dual polarity GRAPPA. Fuzzy Ripple artifacts in the shorter echos spacing images are mitigated, but still visible.

C) depicts the same protocols with 3D-EPI. Due to its different M_z steady-state behavior, 3D-EPI has an inherently higher SNR. 3D-EPI suffers from Fuzzy Ripples, especially for shorter echo spacings.

D) depicts 3D-EPI with 3D-EPI with dual polarity averaging. It can be seen that Fuzzy Ripples are mitigated across echo spacings.

Acquisition parameters of data presented here are mentioned in methods section 3.4.

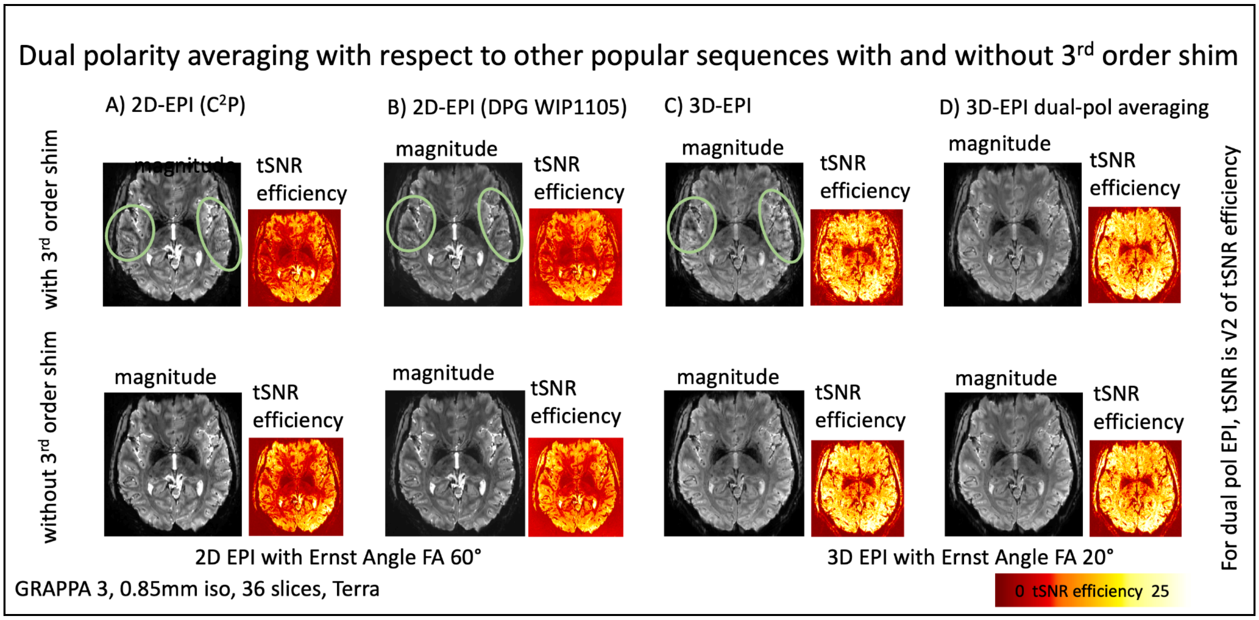

Fig. S6: further reproduced results of Fig. 7 with and without third order shims .

All sequences are used with the same resolution, and acceleration parameters. Echo spacing is constant for results in each row, respectively.

A) depicts the CMRR multiband sequence with these protocols. Fuzzy Ripple artifacts are visible when the 3rd order shim is connected. Without the third order shim, Fuzzy Ripples are not visible.

B) depicts the MGH simultaneous multi slice sequence of this protocol with its option of dual polarity GRAPPA. Fuzzy Ripple artifacts are solely visible with connected 3rd order shims.

C) depicts the same protocols with 3D-EPI. Due to its different M_z steady-state behavior, 3D-EPI has an inherently higher SNR. 3D-EPI suffers from Fuzzy Ripples solely when 3rd order shims are connected.

D) depicts 3D-EPI with 3D-EPI with dual polarity averaging. Neither of the two settings exhibit detectable Fuzzy Ripple artifacts. This means that as soon as the 3rd order shims are disconnected, dual-polarity averaging does not seem to improve the data quality significantly further.

Acquisition parameters of data presented here are mentioned in methods section 3.4.

Generalizability of the mitigation strategy to fMRI applications across resolutions, TRs, and lower brain areas

Imaging the Amygdala and the Cerebellum

Six participants were scanned to evaluate the feasibility of imaging small brain areas at 0.8mm resolution using dual-polarity averaging. Scanning was conducted with 0.8 mm isotropic resolution, partial Fourier 6/8, GRAPPA 3, and 32-channel Rx Nova coils, with a total acquisition time of 14 minutes per functional experiment on a SIEMENS 7T Terra scanner equipped with SC72 gradient sets. Full protocol details are available here: https://github.com/layerfMRI/Sequence_Github/tree/master/low_brain.
Functional tasks included 14 repetitions of 30-second blocks of activation and rest. The amygdala was activated by presenting fearful faces versus objects, and the cerebellum was activated using finger tapping tasks. Results are presented in Fig. 7C and Fig. S8C.

Experiments to exemplify limits of sampling speed at 0.6 mm resolutions

To obtain high-resolution fMRI connectivity datasets with whole-brain coverage, we used the Next Gen 7T scanner (Terra-impulse edition) with its advanced Impulse gradient system (Siemens) featuring a slew rate of 900 T/m/s and a maximum gradient of 200 mT/m. The experiments employed a 64-channel Rx, 8-channel Tx coil (MR CoilTec) [Gunamony 2022]. Three participants were scanned at 0.64 mm resolution with 3D-EPI. To cover the whole brain with 180 slices in a reasonable 11-second acquisition time, we used aggressive GRAPPA acceleration (3 x 2). In the low-SNR regime of such small volume voxels, such high acceleration GRAPPA unaliasing is limited by artifacts from EPI phase inconsistencies. We investigated whether dual-polarity averaging can mitigate these artifacts. Additional imaging parameters included: multi-shot 2 segmentation in the in-plane axis, TE = 20 ms, echo spacing = 0.69 ms, BW = 1592 Hz, FOV = 200 x 200 mm, matrix size 314 x 314. Full protocol details are available here: https://github.com/layerfMRI/Sequence_Github/blob/master/dual-polarity/FeinbergatronWholeBrain_invivo20220710.pdf. We utilized 15 minutes of movie clips from the 7T HCP study (MOVIE1) to explore advanced fMRI methodologies. Results are presented in Fig. 8B.

Functional acquisition contrast

For all functional runs, we combined the sequence with VASO imaging. This involved a global inversion pulse that saturates the blood signal every other TR to localize fMRI signal changes without contamination from large draining veins (Huber 2014).

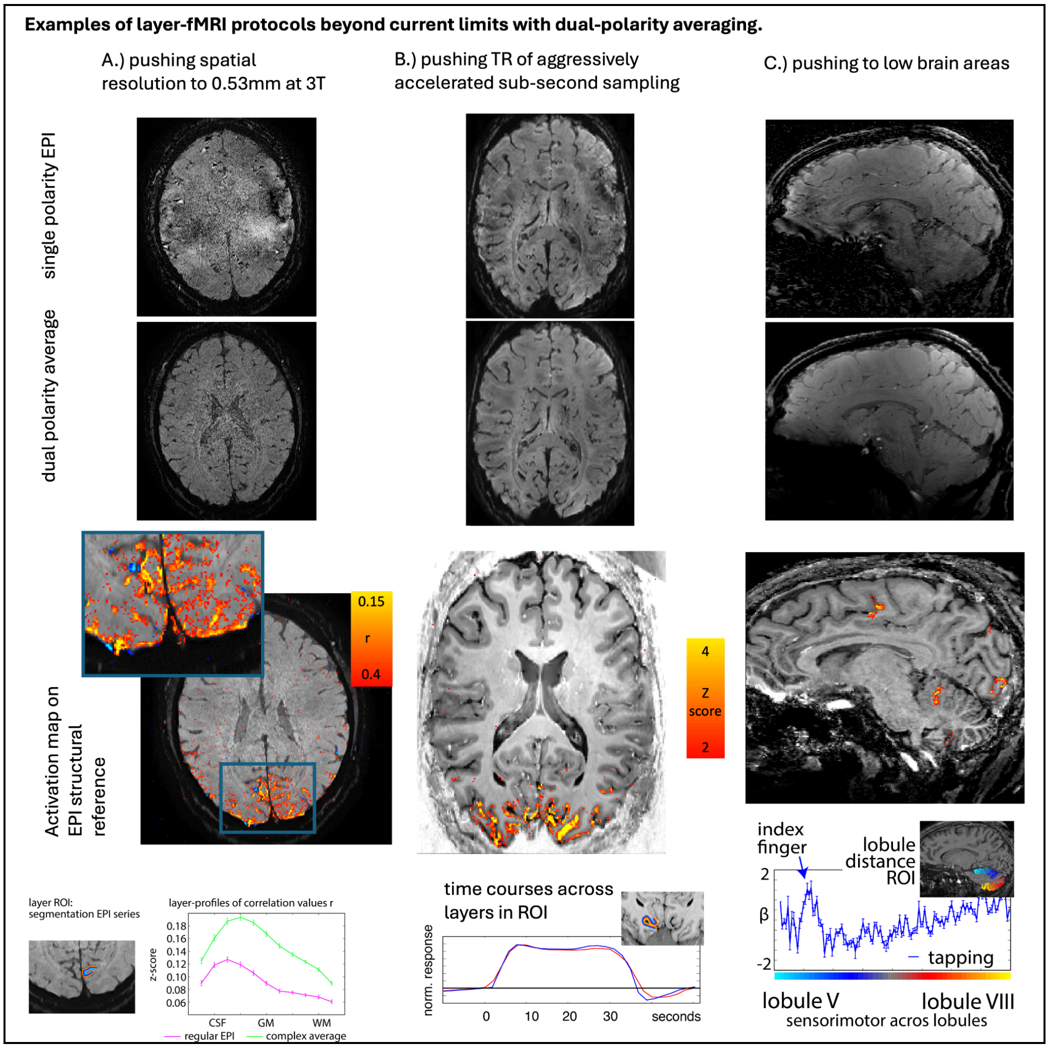

Fig. S7: Examples of high resolution protocols pushing the limits of conventional protocols.

The individual panels illustrate that achieving high spatial resolution, rapid sampling (through acceleration), and imaging of lower brain areas is challenged by Fuzzy Ripples (as demonstrated in Figure 1). However, dual polarity averaging can mitigate these challenges, enabling the extension beyond the current limitations of conventional layer-fMRI protocols.

A) *3T Prisma, 3D-EPI with GRAPPA 3, 15-minute movie-watching paradigm, resolution of 0.53 mm. Acquisition parameters of the data shown here are described in methods section 3.2.*

B) *7T Terra, 3D-EPI, GRAPPA 3, three times 14 min checkerboard, resolution 0.82mm,* TR_{vol}*=0.98s for 14 slices.*

C) *7T Terra, 3D-EPI, GRAPPA 3, three averages of a 12-minute finger-tapping experiment, resolution of 0.82 mm. Acquisition parameters of the data shown here are described in methods section 3.7.*

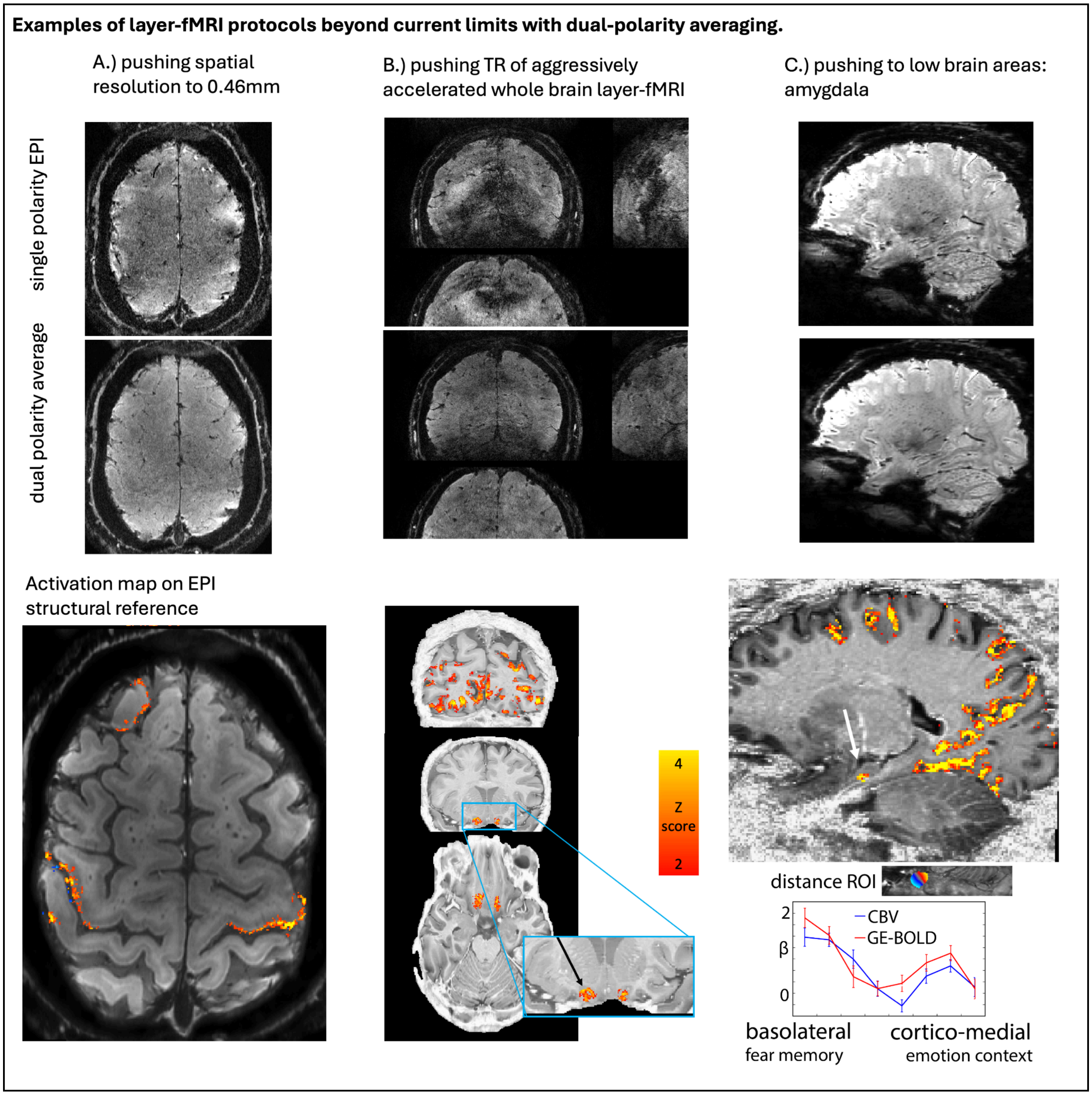

Fig. S8: reproduced results of Fig. 8: Examples of high resolution protocols pushing the limits of conventional protocols.

The individual panels show that pushing to high spatial resolution, to fast sampling (by means of acceleration), and to lower brain areas is challenged by Fuzzy Ripples (as shown in Fig. 1). Dual polarity averaging can mitigate these challenges, thus, allowing to overcome the current limits of conventional layer-fMRI protocols.

***A)** 7T Terra, 3D-EPI with GRAPPA 3, 6 fold segmentation, four runs of 12 min finger tapping, resolution of 0.46 mm.*

***B)** Next Gen 7T, 3D-EPI, GRAPPA 8, three 15-minute movie-watching sessions, resolution of 0.64 mm, TR=11.5 s for 314x314x180 voxels. Acquisition parameters of the data shown here are described in methods section 3.8.*

***C)** 7T Terra, 3D-EPI, GRAPPA 3, three times 15 min emotional faces vs. objects, resolution 0.82mm, Sagittal for deeper brain areas.*

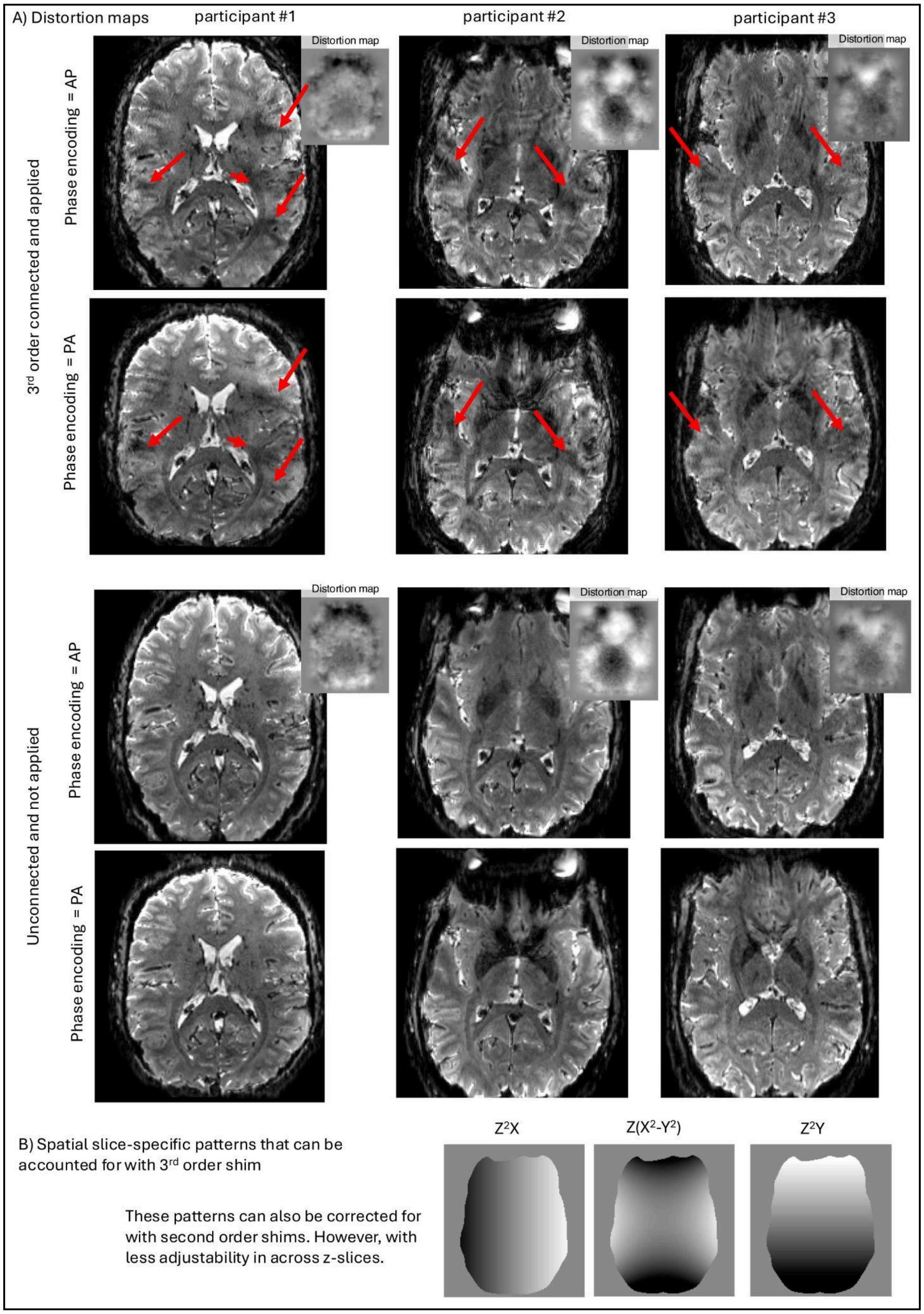

Supplementary Fig. 9

Geometrical distortions of axial EPI slabs with and without 3rd order shims. It can be seen that the distortions are substantial either way. Qualitatively they are quite similar. The average voxels-shift is 1.5mm(±0.5mm) with the 3rd order shim and 1.8mm (±0.7mm) without the 3rd order shim, respectively. It can be seen that the Fuzzy Ripple artifacts (red arrows) are solely visible with the 3rd order shim plugged in.

The geometric distortions can be better appreciated on dynamic figures of animated GIFs here: [*https://github.com/layerfMRI/Sequence_Github/blob/master/Terra_protocolls/3rd_order_shim/distortions.gif*](https://github.com/layerfMRI/Sequence_Github/blob/master/Terra_protocolls/3rd_order_shim/distortions.gif)

Interaction of artifacts in DPG and third order shims as a 2 by 2 design of DPG and third order shim.

It can be qualitatively seen that EPI image integrity is compromised independent of whether DPG is applied or not. Artifacts are visible with and without DPG in areas of blue ellipses. However, when unplugging the 3rd order shim, these artifacts are resolved for both protocols, with and without DPG.

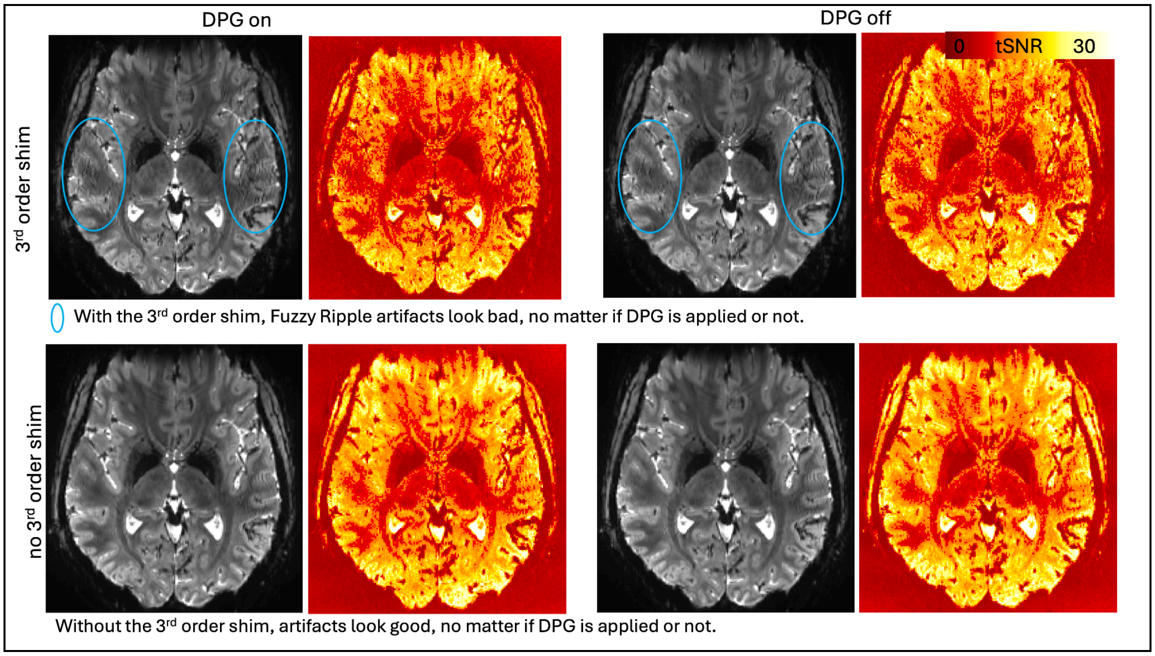

Fig. S10: Results of the DPG WIP 1105 sequence at 0.8mm isotropic with echo spacing of 1.26ms. This figure shows the interaction of artifacts in DPG and third order shims as a 2 by 2 design. It can be qualitatively seen that EPI image integrity is compromised independent of whether DPG is applied or not. Artifacts are visible with and without DPG in areas of blue ellipses. However, when unplugging the 3rd order shim, these artifacts are resolved for both protocols, with and without DPG. Supplementary Figure S11 presents a reproduction of the results shown here.

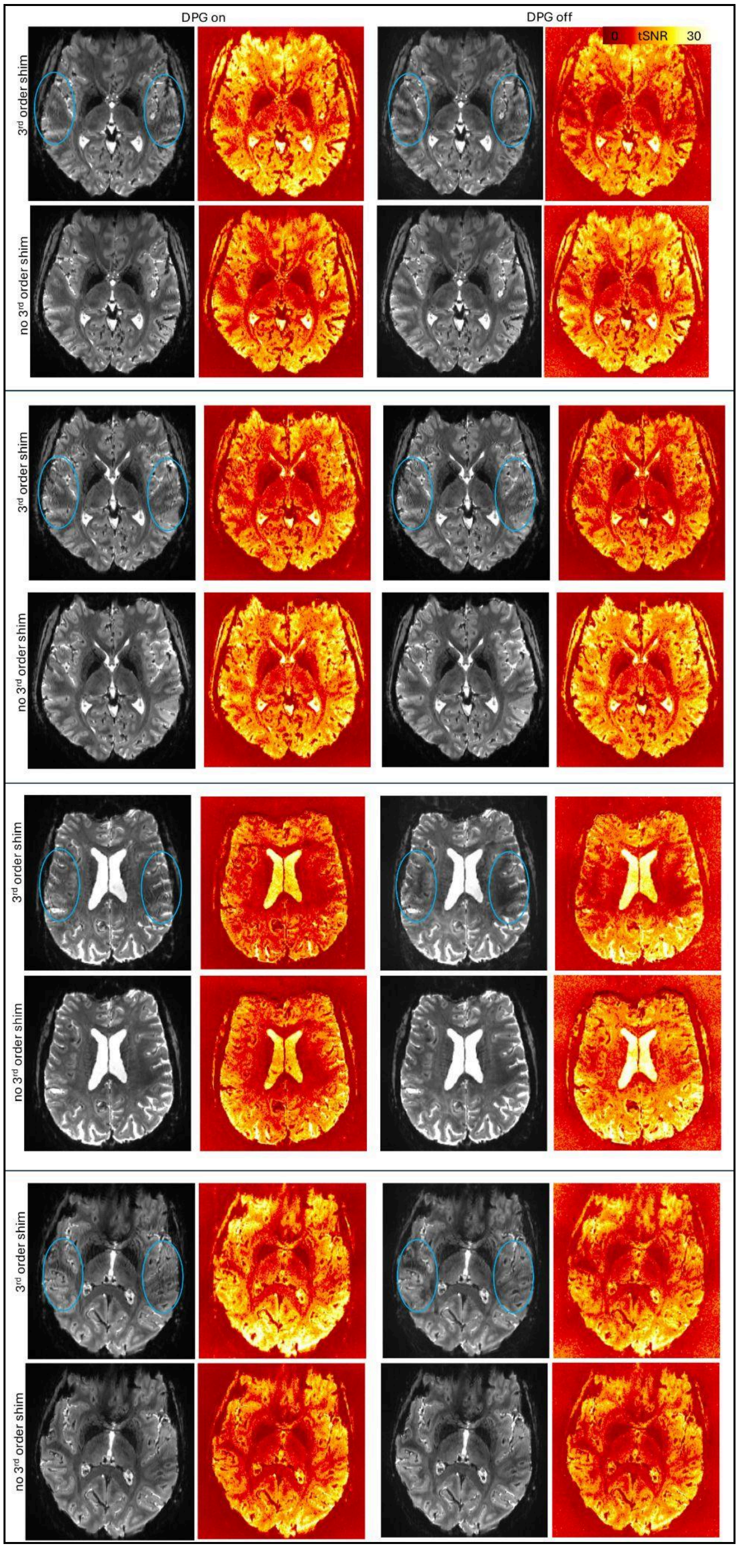

Supplementary Fig. 11, reproducibility of Fid. S10 across participants.

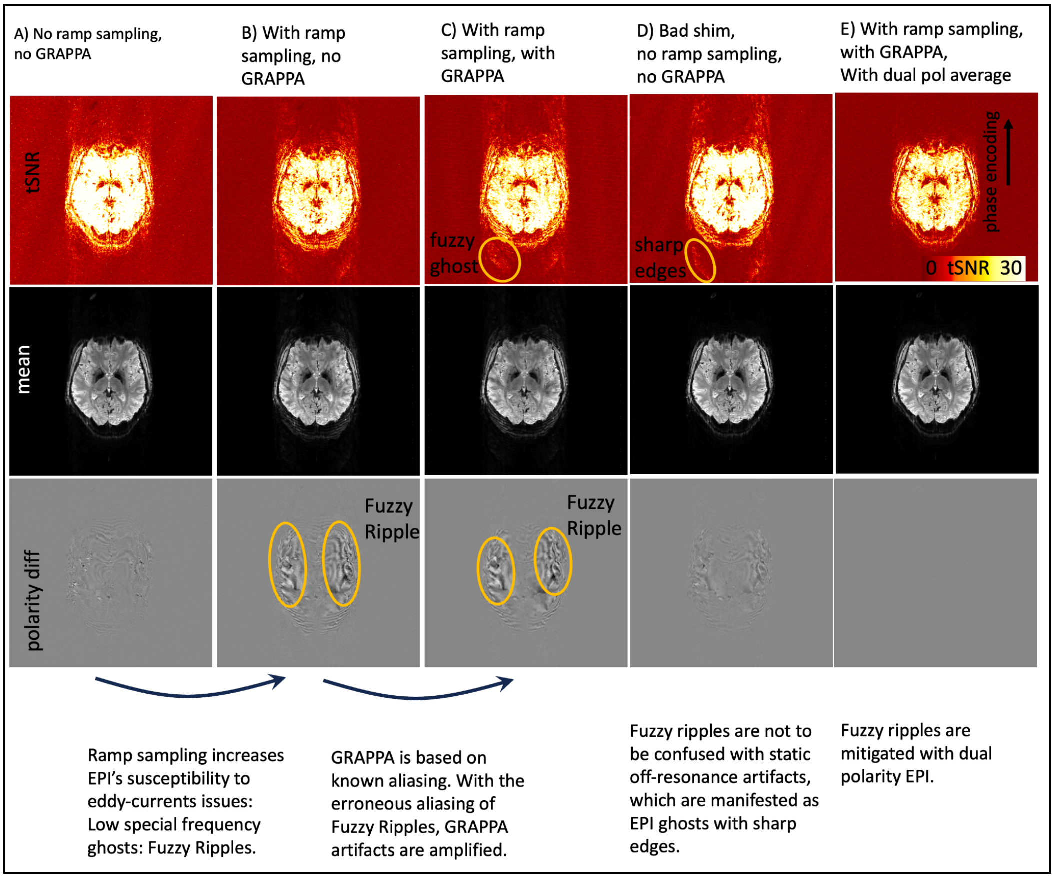

Fig. S12, reproducibility check of Fig. 4 and Fig. S2.

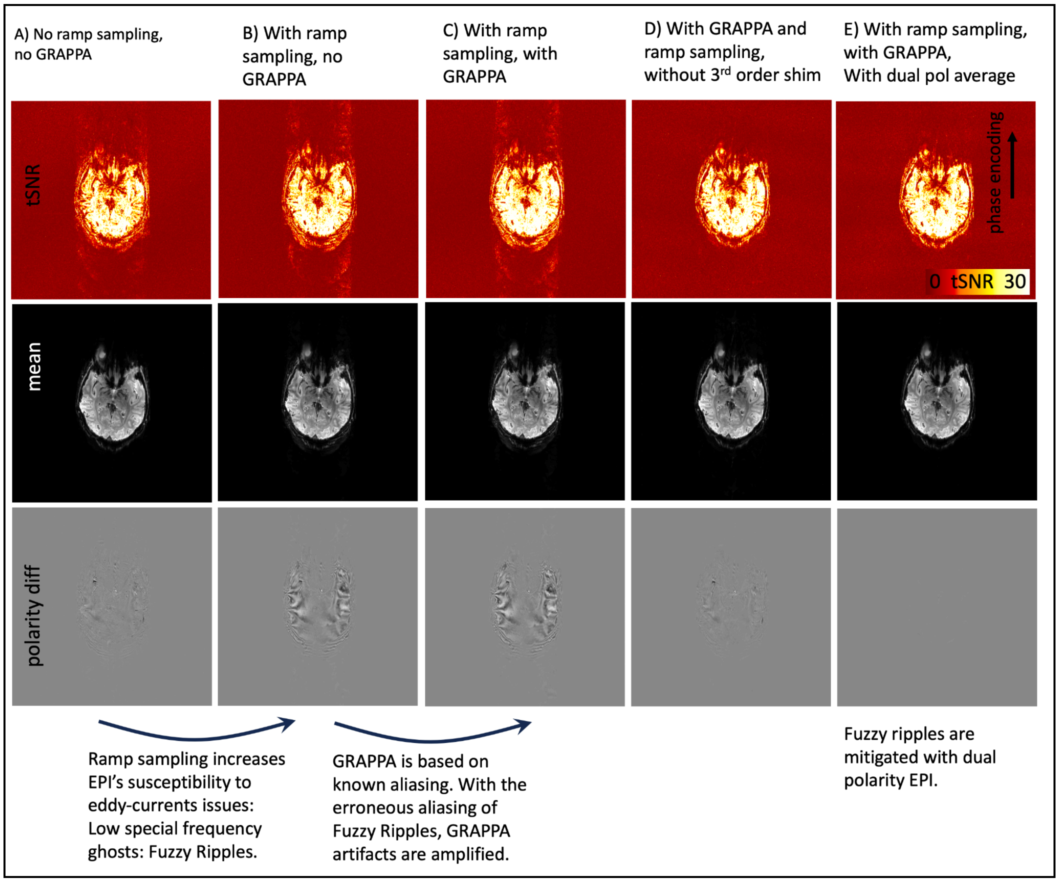

Fig. S13, reproducibility check of Fig. 4, S2, and S12.

Here we also included column D) to show that Fuzzy Ripples are reduced when disconnecting the third order shim. Residual Fuzzy Ripples in panel D are presumably arising from short term eddy currents as discussed in Fig. 3.
